# Serotyping and Antimicrobial Resistance Profile of Enteric Nontyphoidal *Salmonella* Recovered from Febrile Neutropenic Patients and Poultry in Egypt

**DOI:** 10.3390/antibiotics10050493

**Published:** 2021-04-26

**Authors:** Reem A. Youssef, Ahmad M. Abbas, Ahmed M. El-Shehawi, Mona I. Mabrouk, Khaled M. Aboshanab

**Affiliations:** 1 Department of Microbiology, Egyptian Drug Authority (EDA), P.O. Box 11553, Giza 11553, Egypt; reemyoussef9@gmail.com (R.A.Y.); monambrk@gmail.com (M.I.M.); 2 Department of Microbiology and Immunology, Faculty of Pharmacy, Ain Shams University, Organization of African Unity St., P.O. Box 11566, Cairo 11566, Egypt; ahmad.abbas99@pharma.asu.edu.eg; 3 Department of Biotechnology, College of Science, Taif University, P.O. Box 11099, Taif 21944, Saudi Arabia; a.elshehawi@tu.edu.sa

**Keywords:** nontyphoidal *Salmonella*, serotyping, antimicrobial resistance, virulence genes, ERIC PCR

## Abstract

A total of 300 human fecal samples were collected from febrile neutropenic patients suffering from severe gastroenteritis, followed by identification and serological characterization of recovered isolates. Fifty nontyphoidal *Salmonella* (NTS) serovars were recovered. A total of serologically identified 50 NTS serovars recovered from poultry of the same geographical area and during the same period as well as one standard strain *S.* Poona were supplied by the Bacterial Bank of Animal Health Research Institute of Egypt. Antibiogram analysis revealed that the human and poultry serovars exhibited similar antimicrobial resistance patterns against 28 different antimicrobial agents, particularly against ampicillin, cefotaxime, oxytetracycline, and erythromycin. Plasmids harboring *bla*_CTX-m_, *bla*_SHV_, *bla*_TEM_, and *aac*(6’)-Ib were detected in 11 (22%) and 8 (16%) of human and poultry serovars, respectively. Molecular detection of the most clinically relevant virulence genes and analysis of the associated virulence genotypes proved that the human (*n* = 11) and poultry serovars (*n* = 12) shared 11 genotypes. Enterobacterial repetitive intergenic consensus PCR analysis revealed that human and poultry serovars were clustered together in 3 out of the 4 clusters with a similarity index ranged from 0.15 to 1. Since poultry are usually consumed by humans, the presence of resistant bacteria harboring transmissible genetic elements is of great health concern.

## 1. Introduction

Nontyphoidal *Salmonella* (NTS) is a worldwide cause of food-borne illness in humans, causing salmonellosis [1]. In humans, salmonellosis is transmitted through the ingestion of contaminated food of animal origin (mainly eggs, meat, poultry, and milk). However, other foods, such as green vegetables contaminated by manure, have been involved in its contraction [2]. The feco-oral route may also cause person-to-person transmission [3]. The disease is characterized by acute onset of fever, abdominal pain, diarrhea, nausea, and, to a lesser extent, vomiting. The incubation period of the disease is 12–72 h and illness last for 2–7 days [4].

In most cases, symptoms of salmonellosis are mild, and patients will recover without specific treatment. However, in some cases, mainly children and elderly patients, the resulting dehydration may become severe and fatal [5]. Although most salmonellosis infections are mild, they account for about 93.8 million foodborne illnesses and 155,000 deaths per year worldwide, making infections a great concern to public health [6]. More than 2500 *Salmonella* serovars have been identified, most of which belong to *Salmonella enterica* subsp. enterica, which is responsible for most *Salmonella* infections in humans [7]. *Salmonella* infections could be invasive, requiring efficient antibiotic therapy [8].

*Salmonella* virulence is due to presence of chromosomal and plasmid genes. Chromosomal genes are large gene cassettes, called *Salmonella* pathogenicity islands (SPIs), which code about 60 genes that account for certain interactions with the human or animal hosts [9]. Plasmid genes can transfer antimicrobial resistance between bacteria, which can lead to emergence of multidrug resistant (MDR) bacteria and to increasing bacterial virulence, which is a great foodborne risk to human health [10]. The emergence of multidrug resistant (MDR) *Salmonella* serovars exerts a dramatic influence on the efficacy of antibiotic therapy. Increasing prevalence of MDR strains can cause a rise in mortality rates of *Salmonella* infections [11]. The broad prevalence of antimicrobial resistance among nontyphoidal *Salmonella* imposes a great threat to public health since these pathogens are mostly food-borne. 

Hence, appropriate measures and new guidelines should be established to address the rational use of antibiotics and to prevent their abuse, since they represent a major healthcare challenge. Therefore, the aim of our study was to isolate and identify the most common *Salmonella* serovars recovered from febrile neutropenic patients suffering from severe gastroenteritis, collected from different microbiological labs in Egypt, followed by phenotypic and molecular analysis of the antimicrobial resistance profiles, and to compare those serovars with those of poultry origin recovered from the same geographical area and during the same period.

## 2. Results

### 2.1. Isolation and Serological Identification

Out of 300 human fecal samples that were bacteriologically examined, 56 samples were positive for *Salmonella* in a percentage of 18.66%, of which 50 isolates were NTS (16.66%), and 6 isolates were typhoidal *Salmonella* (2%). Serological identification of the recovered *Salmonella* isolates showed that they belonged to 15 different serovars, which were *S.* Typhimurium (10; 17.85%), *S.* Enteritidis (8; 14.28%), *S.* Lumberhurst (8; 14.28%), *S.* Tumodi (4; 7.14%), *S.* Tsevie (4; 7.14%), *S.* Butontan (3; 4.28%), *S.* Anatum (3; 4.28%), *S.* Taksony (2; 4.28%), *S.* Hull (2; 4.28%), *S.* Agama (2; 4.28%), *S.* Dublin (2; 4.28%), *S.* Blegdam (2; 4.28%), *S.* Paratyphi A (3; 4.28%), *S.* Paratyphi C (2; 3.57%), and *S.* Paratyphi B (1; 1.78%). The prevalence of NTS serovars isolated from humans is shown in Table 1.

The 50 poultry isolates and the standard strain (*S*. Poona) were provided serologically identified by AHRI. The poultry serovars were as shown in Table 2.

### 2.2. Antibiogram Pattern

Antibiogram pattern for human serovars revealed that most of them were sensitive to ciprofloxacin (100%), imipenem (100%), norfloxacin (96%), and cefoperazone/sulbactam (92%), followed by trimethoprim/sulphamethoxazole (84%), colistin/sulfamethazine (82%), and flumequine (82%). On the other hand, all serovars showed 100% resistance to clindamycin, erythromycin, rifampicin, cefotaxime, amoxicillin, tobramycin, amoxicillin/clavulanic acid, and ampicillin, followed by ampicillin/sulbactam (98%) and lincomycin (92%). Various sensitivity patterns were exhibited with the rest of the used antibiotics (Table 3).

**Figure 1:**
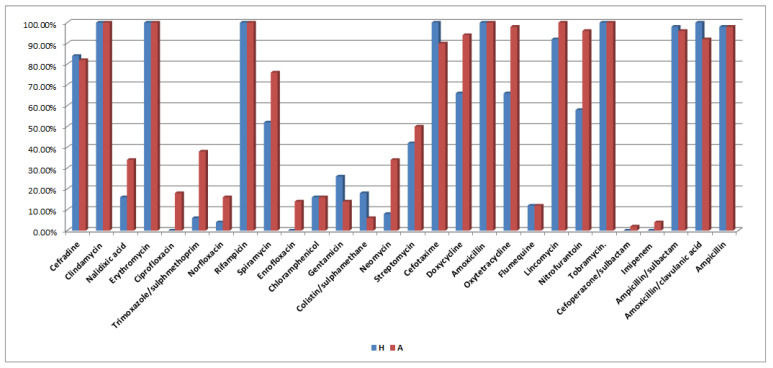
Antimicrobial resistance patterns of human (*n* = 50) and poultry (*n* = 50) *Salmonella* serovars against 28 various antimicrobial agents.

For poultry serovars, results reveal that most serovars were sensitive to imipenem (96%), cefoperazone/sulbactam (94%), and colistin/sulfamethazine (92%), followed by ciprofloxacin (80%). On the other hand, all serovars showed 100% resistance to clindamycin, erythromycin, rifampicin, amoxicillin, lincomycin, and tobramycin, followed by ampicillin and oxytetracycline (both with 98%), ampicillin/sulbactam and nitrofurantoin (both with 96%), doxycycline (94%), amoxicillin/clavulanic acid (92%), cefotaxime (90%), and cefradine (82%). Variable sensitivity results were recorded for other used antibiotics (Table 3). The detailed antibiogram analysis for each human and poultry serovar is shown in Tables S1 and S2 (supplementary data), respectively. The antibiogram analysis of human (*n* = 50) and poultry (*n* = 50) *Salmonella* serovars against 28 various antimicrobial agents is depicted in Figure 1.

*S.* Poona standard serovar was sensitive to ciprofloxacin, trimethoprim/sulphamethoxazole, norfloxacin, enrofloxacin, gentamicin, colistin/sulfamethazine, neomycin, flumequine, cefoperazone/sulbactam, and imipenem, while it was resistant to cefradine, clindamycin, erythromycin, rifampicin, streptomycin, cefotaxime, doxycycline, amoxicillin, oxytetracycline, lincomycin, tobramycin, ampicillin/sulbactam, amoxicillin/clavulanic acid, and ampicillin (Table 3). Human, poultry, and the standard strain were found to be 100% MDR by 3 or more of the selected antibiotics, as shown in Table S3.

### 2.3. Plasmid Profile Analysis

Results reveal that, in addition to the standard strain, 19 (63.3%) out of the 30 tested serovars (66.67%) that were ampicillin resistant showed plasmid DNA. Eleven human serovars (22%; *n* = 50), including H2, H3, H4, H5, H11, H12, H13, H22, H28, H31, and H36 and eight (16%; *n* = 50) poultry serovars, including A52, A63, A75, A77, A78, A79, A81, and A83, carried plasmid DNA (Tables S4 and S5 and Figure S1A–C). The antimicrobial resistance genes detected in the human and poultry Salmonella serovars up on using plasmid DNA of each serovar as a PCR template is shown in Table 4.

### 2.4. Detection of Certain Virulence Genes

**Figure 2:**
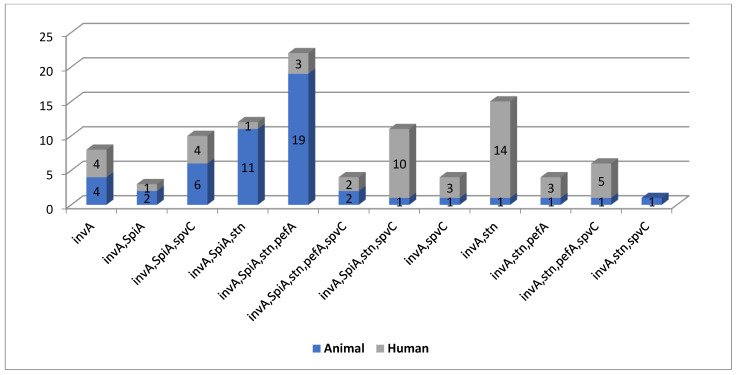
Shared virulence genotypes among human and poultry NTS serovars as well as prevalence of each genotype.

Out of the 50 isolated human serovars, 100% were positive for *inv*A gene, 46% for both *spi*A and *stn*, 40% positive for *pef*A, and 42% were positive for *spv*C. Additionally, out of 50 poultry serovars, 100% were positive for *inv*A gene, 82% were positive for *spi*A, 74% were positive for *stn*, 48% positive for *pef*A, and 24% were positive for *spv*C. *S.* Poona standard strain was positive for all virulence genes (Figures S2–S5). Table 5 and Table 6 show various virulence genotypes among human and poultry serovars. Figure 2 reveals shared virulence genotypes among human and poultry *Salmonella* serovars as well as prevalence of each genotype. Percentages of detection of different virulence genes among tested serovars are shown in Table 7 and Tables S4 and S5 (supplementary data).

### 2.5. Enterobacterial Repetitive Intergenic Consensus (ERIC)-PCR

**Figure 3:**
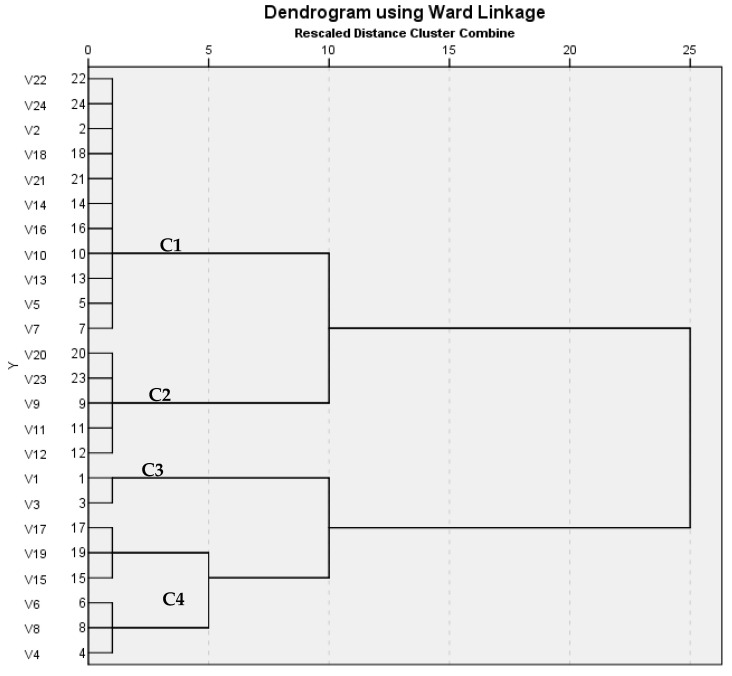
Dendrogram of the ERIC-PCR for the 24 samples (codes V1–V24) generated by the unweighted pair group method with arithmetic average (UPGMA) and Ward’s hierarchical clustering routine. V1 (*S.* Typhimurium H4), V2 (*S.* Enteritidis H14), V3 (*S.* Lumberhurst H23), V4 (*S.* Tumodi H28), V5 (*S.* Tesive H32), V6 (*S.* Anatum H38), V7 (*S.* Taksony H42), V8 (*S.* Hull H43), V9 (*S.* Agama H45), V10 (*S.* Dublin H48), V11 (*S.* Blegdam H49), V12 (*S.* Butontan H35), V13 (*S.* Chester A71), V14 (*S.* Infantis A72), V15 (*S.* Kentucky A67), V16 (*S.* Virchow A75), V17 (*S.* Newport A85), V18 (*S.* Nitra (dog) A80), V19 (*S.* Montevideo A88), V20 (*S.* Gueuletapee A92), V21 (*S.* Kottbus A95), V22 (*S.* Sekondi A97), V23 (*S.* Newlands A100), V24 (*S.* Gallinarum A98).

As shown in Figure S6, the ERIC-PCR analysis of the 24 (12 human and 12 poultry) serovars yielded different patterns consisting of 1–6 bands by which the serovars were grouped into 4 clusters (C1 to C4) (Figure 3). Similarity index between all the 24 serovars was calculated using the Jaccard/Tanimoto Coefficient and the number of intersecting elements, and it ranged from 0.15 to 1 (Table 8).

## 3. Discussion

*Salmonella* is a major cause of food-borne illness in humans, and salmonellosis is mainly contracted through ingestion of contaminated food of poultry origin including, undercooked meat, eggs, and other poultry-derived food [12]. The *Salmonella* organisms can survive in undercooked food and then be transmitted to humans, causing pathological conditions, such as gastroenteritis and fever in humans, which can be associated with significant clinical consequences and complications. Additionally, the antimicrobial resistant genotypes can be carried on transferrable genetic elements such as plasmids or integrons, which can then be transferred from poultry to human serovars and to the bacterial flora—a major consequence of ingestion of undercooked food containing living poultry *Salmonella* serovars. In our study, bacteriological examination of 300 human fecal samples collected from febrile neutropenic patients suffering from severe gastroenteritis revealed that NTS represented 16.66% of the recovered isolates (50 isolates), while 6 isolates (2%) were typhoidal *Salmonella*. Most of the recovered NTS isolates belonged to *S.* Typhimurium (10; 17.85%) and *S.* Enteritidis (8; 14.28%), followed by *S.* Lumberhurst (8; 14.28%) serovars. These results corroborate those reported by Ahmed et al. [13], who found that the isolation rate from stool swabs was 18.7% of *Salmonella* spp. On the other hand, Diab et al. [12] isolated *Salmonella* from stool samples in a percentage of 4.4% and also serotyped these isolates as *S.* Typhimurium and *S.* Enteritidis. Adesoji et al. [14] isolated *Salmonella* in a percentage of 60% from stool samples of diarrheic patients.

The emergence of antibiotic resistance poses a significant public health risk. Antibiotic consumption and the emergence/dissemination of resistant strains in hospitals and intensive care units have been clearly linked in epidemiological studies. Antibiotic therapy is an important factor that induces alteration of gut microbiota composition (called dysbiosis), leading to pathology, including asthma and infectious disease [15]. Gut microbiota is necessary for proper intestinal tract development as well as immune and nervous system maturation. In fact, an intact, fully developed gastrointestinal (GI) tract microbiota also protects the host from pathogenic microorganism invasion [16,17,18] via a highly complex set of events known as colonization resistance [19,20]. 

Microorganisms coexist in the same ecological niche, i.e., microbiotas, but also a continuum of microorganism exchange occurs between humans, animals, and the environment. For example: yeasts can be found in all environments and have been described as effective antagonists of a variety of plant pathogens [21]. Additionally, increased “contact” between animals and humans increases the risk of infection and the spread of antimicrobial resistance (AMR) traits. As a result, the possibility of reverse zoonosis, as well as the establishment of animal reservoirs that maintain the infection cycle and AMR spread, is now a growing concern. Antimicrobial resistance of animal origin, which poses a direct and/or indirect threat to human health, especially for extended spectrum beta-lactamase (ESBL) Gram-negative bacteria. [22].

The antibiogram pattern for human serovars revealed all serovars to be 100% resistant to clindamycin, erythromycin, rifampicin, cefotaxime, amoxicillin, tobramycin, amoxicillin/clavulanic acid, and ampicillin, followed by ampicillin/sulbactam (98%) and lincomycin (92%). For poultry serovars, results reveal that all serovars showed 100% resistance to clindamycin, erythromycin, rifampicin, amoxicillin, lincomycin, and tobramycin, followed by ampicillin and oxytetracycline with 98%, ampicillin/sulbactam and nitrofurantoin with 96%, doxycycline (94%), amoxicillin/clavulanic acid (92%), cefotaxime (90%), and cefradine (82%). Additionally, *S.* Poona standard strain was resistant to cefradine, clindamycin, erythromycin, rifampicin, streptomycin, cefotaxime, doxycycline, amoxicillin, oxytetracycline, lincomycin, tobramycin, ampicillin/sulbactam, amoxicillin/clavulanic acid, and ampicillin. Interestingly, most of the studies conducted on the recovery of human and poultry *Salmonella* serovars were found to be resistant to clindamycin, erythromycin, rifampicin, amoxicillin, tobramycin, ampicillin, ampicillin/sulbactam, amoxicillin/clavulanic acid, cefotaxime, lincomycin, and cefradine. The similarity in the resistance patterns between human and poultry serovars suggests that they could be of the same source, pointing out the zoonosis of this microorganism. Moreover, strong evidence was obtained when the standard strain that was used showed the same resistance patterns.

Moreover, our findings nearly coincide with those obtained by Diab et al. [12] using 12 antimicrobials: 86.4% were resistant to oxytetracycline and streptomycin, followed by neomycin and erythromycin (77.3%) and norfloxacin and ampicillin (68.2%). On the contrary, 90.9% were sensitive to gentamicin. In addition, Adesoji et al. [14] mentioned that *Salmonella* spp. were 100% resistant to amoxicillin and ampicillin. Furthermore, Voss-Rech et al. [23] isolated NTS of poultry and human origin in Brazil and found that NTS of poultry origin showed high resistance to sulfonamides (44.3%), nalidixic acid (42.5%), and tetracycline (35.5%), while in those of human origin, the resistance occurred mainly for sulfonamides (46.4%), tetracycline (36.9%), and ampicillin (23.6%). Meanwhile, Singh [24] stated that *Salmonella* isolated from poultry were resistant to ampicillin and tetracycline but not to enrofloxacin. Additionally, Ouali et al. [25] stated that clinical isolates from a tertiary referring hospital were sensitive to trimethoprim–sulfamethoxazole (72%), chloramphenicol (64%), ampicillin (48%), gentamicin (44%), and ciprofloxacin (2%). Plasmid profile analysis showed that out of the 30 ampicillin-resistant serovars, 19 (63.3%) had plasmid, as did the standard strain. These findings are nearly similar to those of the work of McMillan et al. [10], which stated that more than 80% of antibiotic resistance genes were located within a plasmid and that many different plasmids were involved in antibiotic resistance in *Salmonella* among food animals. In addition, Dos Santos et al. [26] mentioned that in *S.* Typhimurium, most of the virulence factors encoding genes are located in *Salmonella* pathogenicity islands (SPI). However, the other virulence genes were located in virulence plasmids (pSLT), particularly in the *spv*-operon containing plasmids [26]. Plasmid genes have the ability to transfer antimicrobial resistance between bacteria, which can lead to the presence of multidrug resistant (MDR) bacteria, making them more dangerous to human health [10]. In our study, plasmids harboring *bla*_CTX-m_, *bla*_SHV_, *bla*_TEM_, and *aac*(6′)-Ib were detected in 11 (22%) and 8 (16%) of human and poultry serovars, respectively. Since poultry are usually consumed by humans, the presence of resistant bacteria harboring transmissible genetic elements is of great health and medical importance.

Out of the 50 isolated human serovars, 100, 26, 40, and 42% were positive for *inv*A gene, both *spi*A and *stn*, *pef*A, and *spv*C, respectively. Additionally, out of 50 poultry serovars, 100, 82, 74, 48, and 24% were positive for *inv*A gene, *spi*A, *stn*, *pef*A, and *spv*C, respectively. The *S.* Poona standard strain was positive for all tested virulence genes. Molecular detection of the most clinically relevant virulence genes and analysis of the associated virulence genotypes proved that the human (*n* = 11) and poultry serovars (*n* = 12) shared 11 genotypes. These findings are in agreement with those of a previous study [27] that reported that, out of 33 NTS, *inv*A was positive for 28 isolates, of which 89.3% patients were febrile. This study also revealed a positive correlation between the *inv*A gene and febrile illness, and therefore highlights the importance of *inv*A as an important marker for bloodstream invasion. Kim et al. [28] reported that virulence genes profiling showed that *inv*A and *spi*A were found in all antimicrobial-resistant NTS isolates, while *pef*A was found in 55% of the resistant NTS isolates. Moreover, Abraham and his coworkers [29] mentioned that *inv*A gene was detected in all isolates. Furthermore, 94% of the isolates from sheep showed an abundance of the *spv*C gene. However, the *pef*A gene was detected in 18 isolates from chicken and 1 isolate from sheep. Moreover, a previous study [30] detected the presence of the *stn* gene in 85.7% isolates of *S.* Enteritidis among 41 collected isolates. 

ERIC-PCR is a highly effective DNA-based typing method for fingerprinting. Epidemiology of *Salmonella* Enteritidis can be studied through using ERIC-PCR analysis [31]. In this study, ERIC PCR was performed on 12 different human serovars, which were compared with 12 different poultry serovars; the analysis showed different patterns with 1–6 band, a dendrogram revealed four clusters, and the similarity index for them showed similarity that ranged from 0.15 to 1. Similar findings were reported by Kim et al. [28], which stated that distribution of profiles among serotypes revealed that different serotypes had similar fingerprinting patterns. Additionally, Khakzad et al. [32] stated that most isolated strains were relevant to *Salmonella* Enteritidis, and a dendrogram study showed that the bacteria were grouped in one cluster in a dendrogram that all 37 strains were put into—a large cluster of *Salmonella*’s type which was divided into two clusters: *Salmonella* Enterica and Bongori. Furthermore, Saravanan et al. [33] mentioned that ERIC-PCR was used to determine the degree of variation between the isolates. All the isolates produced 8 distinct banding patterns (ERIC 1–8) ranging from 1 to 6 bands, with the fragments ranging from 150 to 2000 bp. Molecular detection of the most clinically relevant virulence genes and analysis of the associated virulence genotypes proved that the human (*n* = 11) and poultry serovars (*n* = 12) shared 11 genotypes. ERIC-PCR analysis revealed that human and poultry serovars clustered in 3 out of the 4 clusters using Jaccard/Tanimoto Coefficient with a similarity index ranged from 0.15 to 1. 

## 4. Materials and Methods

### 4.1. Microorganisms

A total of 300 human fecal samples were collected from febrile neutropenic patients (showing absolute neutrophils count (ANC) less than 500 per mm3 and oral temperature greater than 38 °C for at least 1 h) suffering from severe gastroenteritis; samples were collected from several private laboratories in Cairo, Giza, and Qalubiya governorates during the period from October 2018 to September 2019. 

Samples were collected using sterile swabs, then transferred to an ice box and transported directly to the laboratory. A portion of 10 mL of Muller–Kauffmann tetrathionate/novobiocin broth was added and then incubated at 37 °C for 24 ± 3 h, after which each enrichment culture was streaked onto at least two selective agars for *Salmonella* isolation, mainly xylose lysine decarboxylase (XLD agar) and Brilliant green agar (BG). Identification of the collected isolates was carried out using standard methods of microscopic and biochemical characteristics [34]. The study protocol was reviewed and approved by the institutional ethics committee, Faculty of Pharmacy, Ain Shams University (ENREC-ASU-2018-214), where both informed and written consents were obtained from patients after explaining the purpose of the study. The study was carried out according to the ethical principles stated in the Declaration of Helsinki. A total of serologically identified 50 poultry serovars collected during the same period from poultry farms in Egypt (internal organs: liver, gallbladder, and intestine), large and small animals (fecal swabs), and one standard strain (*S.* Poona) were supplied by the Bacterial Bank of Animal Health Research Institute (AHRI) in Giza, Egypt. The poultry serovars were also collected from Cairo, Giza, and Qalubiya governorates during the period from October 2018 to September 2019. 

### 4.2. Serological Identification

Human isolates that were previously identified as *Salmonella* were subjected to serological identification that was carried out according to White-Kauffman-Le Minor scheme, as described by the Word Health Organization (WHO) Collaborating Centre for Reference and Research on *Salmonella* (WHOCC-Salm) [35]. Identification was accomplished using diagnostic omnivalent (A-O67), polyvalent (A-G), monovalent “O” (O1), polyvalent “H” (phase 1, phase 2), and monovalent H antisera, obtained from DENKA SEIKEN, Japan. Poultry serovars and the standard strain were provided serologically identified by AHRI.

### 4.3. Antibiogram Pattern

Antibiotic sensitivity test was performed using Kirby–Bayer disc diffusion method [36] for all serovars (including human, poultry, and the standard strain) against 28 antibiotics and chemotherapeutic agents belonging to various classes of antibacterial agents acting on Gram-negative bacteria, performed according to CLSI guidelines [37]. The 28 antibiotics were supplied by Oxoid (Hampshire, UK), Himedia (Mumbai, India), Bioanalyse (Ankara, Turkey) and Biorad (Hercules, CA, USA). Multidrug resistance (MDR) was defined as concomitant resistance to ≧3 drug classes of antimicrobials with the same selection, including ampicillin, oxytetracycline, erythromycin, trimethoprim/sulfamethoxazole, chloramphenicol, cefotaxime, and ciprofloxacin.

### 4.4. Plasmid Profile Analysis

Plasmid profile analysis was performed on ampicillin resistant serovars and the standard strain. They were cultured onto Meuller Hinton containing ampicillin 100 µg/mL. Plasmid extraction was carried out from the ampicillin resistance serovars using iNtRON’s DNA-spin™ Kit (iNtODEWORLD, Boston, MA, USA) according to its manufacturer’s manual and that was followed by agarose gel electrophoresis [38] using 1kb marker (Fermentas, Thermo Scientific, Leon-Rot, Germany). The ampicillin resistant serovars were H1 (*S.* Typhimurium), H2 (*S.* Typhimurium), H3 (*S.* Typhimurium), H11 (*S.* Enteritidis), H31 (*S.* Tesive), H36(*S.* Butontan), H22 (*S.* Lumberhurst), H28 (*S.* Tumodi), H4 (*S.* Typhimurium), H12 (*S.* Enteritidis), H5 (*S.* Typhimurium std), STD (*S.* Poona std), H13 (*S.* Enteritidis), A92 (*S.* Gueuletapee), A82 (*S.* Bardo), A96 (*S.* Magherafelt), A67 (*S.* Kentucky), A72 (*S.* Infantis), A74 (*S.* Cremieu), A73 (*S.* Furuch), A65 (*S.* Blegdam), A78 (*S.* Stratford), A77 (*S.* Sanktjohan), A53 (*S.* Enteritidis), A51 (*S.* Typhimurium), A75 (*S.* Virchow), A83 (*S.* Bonariensis), A81 (*S.* Papuana), A79 (*S.* Mississippi), A52 (*S.* Typhimurium), and A63 (*S.* Agama). The plasmid DNA of each serovar was used as a PCR template for detection of the three major extended spectrum β-lactamases (ESBLs), including *bla*_TEM_ coded for TEM extended spectrum β-lactamase, *bla*_SHV_ coded for SHV extended spectrum β-lactamase, and *bla*_CTX-m_ coded for cefotaxime (CTX-M) extended spectrum β-lactamase as well as the *aac*(6′)-Ib coded for aminoglycoside 6′-N-acetyltransferase type Ib ciprofloxacin resistant variant (Table 9). Analysis of the PCR products was carried out using agarose gel electrophoresis [30].

### 4.5. PCR for Detection of the Most Clinically Relevant Virulence Genes

#### 4.5.1. Samples

The 100 serovars (50 human serovars and 50 poultry serovars) and the standard bacterial *Salmonella* strain were cultured onto Meuller Hinton agar and XLD agar.

#### 4.5.2. DNA Extraction

DNA extraction was accomplished using QIAamp DNA Mini Kit (Qiagen, Hilden, Germany) and ethanol 96% (Applichem, Darmstadt, Germany) according to manufacturer’s instructions using specific equipment and apparatuses used for extraction of nucleic acids. 

#### 4.5.3. PCR Conditions

The reaction was conducted using Emerald Amp GT PCR master mix (Takara Bio, Kusatsu City, Japan) and 100 bp DNA molecular weight marker supplied from GeneDireX, Taoyuan, Taiwan). Nine pairs of oligonucleotide primers were supplied from Macrogen, Korea. Their specific sequences and amplification products are shown in Table 9.

#### 4.5.4. Computer Programs

Several computer programs were used for analyses of the tested virulence genes and their encoded proteins. The nucleotides and amino acids sequences of the tested genes were obtained by searching the database (NCBI). The multiple alignments of the nucleotides and amino acids sequences were carried out using Clustal Omega (https://www.ebi.ac.uk/Tools/msa/clustalo/) accessed on 5 April 2021. Analysis of the designed primers was carried out using pDRAW32 program (http://www.acaclone.com/download/install.htm) accessed on 5 April 2021 and Primer-BLAST program on the NCBI database (https://blast.ncbi.nlm.nih.gov/Blast.cgi) accessed on 5 April 2021.

#### 4.5.5. Cycling Conditions of the Primers during PCR

Temperature and time conditions of the primers during PCR were as follows: primary denaturation at 94 °C for 5 min, secondary denaturation at 94 °C for 30 s, and annealing at 55 °C for 1 min for all virulence genes (*inv*A, *spi*A, *spv*C, and *stn*) except for *pef*A (at 54 °C for 1 min), extension at 72 °C for 1 min, followed by final extension at 72 °C for 12 min, repeatedly for 35 cycles for each virulence gene. Agarose gel electrophoreses were conducted according to Sambrook et al. [38].

### 4.6. ERIC PCR

#### 4.6.1. Samples

Twelve different human serovars in comparison to twelve different poultry serovars were selected for ERIC-PCR, as mentioned in Table 10.

#### 4.6.2. DNA Extraction

DNA extraction was performed using QIAamp DNA Mini Kit (Qiagen, Germany) and ethanol 96% (Applichem, Germany) according to manufacturer’s instructions using specific equipment and apparatuses used for extraction of nucleic acids. 

#### 4.6.3. PCR Conditions

The reaction was performed using Emerald Amp GT PCR master mix (Takara Bio, Japan) and 100 bp DNA molecular weight marker supplied from GeneDireX, Taiwan (no. of bands: 12, range: 100–3000 bp). One pair of ERIC primer was supplied from metabion (Planegg, Germany). Its specific sequence and amplification product is shown in Table 11.

#### 4.6.4. Computer Programs

ERIC fingerprinting data were transformed into a binary code depending on the presence or absence of each band. Dendrograms were generated by the unweighted pair group method with arithmetic average (UPGMA) and Ward’s hierarchical clustering routine. Cluster analysis and dendrogram construction were performed with SPSS, version 22 (IBM 2013) [44].

Similarity index (Jaccard/Tanimoto Coefficient and number of intersecting elements) between all samples was calculated using the online tool (https://planetcalc.com/1664/) accessed on April 2021.

#### 4.6.5. Cycling Conditions of the Primers during PCR

Temperature and time conditions during ERIC-PCR were as follows: primary denaturation at 94 °C for 5 min, secondary denaturation at 94 °C for 30 s, and annealing at 52 °C for 1 min, extension at 72 °C for 1 min, followed by final extension at 72 °C for 12 min, repeatedly for 35 cycles. Agarose gel electrophoreses were implemented according to Sambrook et al. [38] with modifications.

## 5. Conclusions

Serotyping and antimicrobial resistance profile of enteric NTS recovered from febrile neutropenic patients and poultry of the same geographical area in Egypt were undertaken. Antibiogram analysis revealed that the human and poultry serovars exhibited similar antimicrobial resistance patterns against 28 tested antimicrobial agents. Plasmids harboring *bla*_CTX-m_, *bla*_SHV_, *bla*_TEM_, and *aac*(6′)-Ib were detected in 11 (22%) and 8 (16%) of human and poultry serovars, respectively. Molecular detection of the most clinically relevant virulence genes and analysis of the associated virulence genotypes proved that the human and poultry serovars shared 11 genotypes. As poultry are usually consumed by humans via ingestion of their meat or come into contact with poultry fecal samples and therefore, get infections by the feco-oral route, the presence of resistant bacteria such as NTS-harbored transmissible genetic elements are a great health issue that should be considered and controlled. Accordingly, preventive measures and new guidelines should be undertaken, particularly in developing countries such as Egypt, to prevent dissemination of the antimicrobial resistance from poultry to human and from human to human thereafter. In addition to the rational use of antibiotics, adequate prophylactic interventions such as immunization programs are strongly recommended.

## Figures and Tables

**Table 1:** The prevalence of NTS serovars isolated from humans.

No. of *Salmonella* Serovars	*Salmonella* Serotypes	No. of Serotypes	Percentage
50	*S.* Typhimurium	10	20%
	*S.* Enteritidis	8	16%
	*S.* Lumberhurst	8	16%
	*S.* Tumodi	4	8%
	*S.* Tesive	4	8%
	*S.* Butontan	3	6%
	*S.* Antum	2	4%
	*S.* Taksony	2	4%
	*S.* Hull	2	4%
	*S.* Agama	2	4%
	*S.* Dublin	2	4%
	*S.* Blegdam	2	4%

**Table 2:** *Salmonella* serovars isolated from poultry.

Serovar	Number of Serotypes	Serovar	Number of Serotypes	Serovar	Number of Serotypes
*S.* Typhimurium	2	*S.* Furuch	1	*S.* Colindal	1
*S.* Enteritidis	5	*S.* Cremieu	1	*S.* Hato	1
*S.* Lumberhurst	1	*S.* Virchow	1	*S.* Montevideo	1
*S.* Tsevie	1	*S.* Kedougou	1	*S.* Farsta	1
*S.* Butontan	1	*S.* Sanktjohan	1	*S.* Kralingen	1
*S.* Anatum	2	*S.* Stratford	1	*S.* Fillmore	1
*S.* Agama	1	*S.* Mississippi	1	*S.* Gueuletapee	1
*S.* Taksony	1	*S.* Nitra	1	*S.* Atakpame	1
*S.* Blegdam	2	*S.* Papuana	1	*S.* Stanly	1
*S.* Kentucky	2	*S.* Bardo	1	*S.* Kottbus	1
*S.* Bouake	2	*S.* Bonariensis	1	*S.* Magherafelt	1
*S.* Chester	1	*S.* Boecker	1	*S.* Sekondi	1
*S.* Infantis	1	*S.* Newport	1	*S.* Gallinarum	1
*S.* Volta	1	*S.* Newlands	1		

**Table 3:** Antimicrobial resistance patterns of human and poultry serovars.

Antimicrobial Agents	Percentage of Resistance%		
	Human Serovars	Poultry Serovars	Standard Strain
Clindamycin (CD 2)	100%	100%	R
Erythromycin (E 15)	100%	100%	R
Rifampicin (RIF 5)	100%	100%	R
Amoxycillin (AMX 10)	100%	100%	R
Tobramycin (TOB 10)	100%	100%	R
Ampicillin (AMP 10)	100%	98%	R
Ampicillin/sulbactam (SAM 20)	98%	96%	R
Amoxacillin/clavulnic acid (AMC 30)	100%	92%	R
Cefotaxime (CTX 30)	100%	90%	R
Lincomycin (L 2)	92%	100%	R
Cefradine (CH 30)	84%	82%	R
Oxytetracycline (O 30)	66%	98%	R
Doxycycline (DO 30)	66%	94%	R
Streptomycin (S 10)	42%	50%	R
Nitrofurantoin (F 300)	58%	96%	I
Spiramycin (SR 100)	52%	76%	I
Nalidixic acid (NA 30)	16%	34%	I
Trimoxazole/sulphmethoprim (COT 25)	4%	38%	S
Norfloxacin (NX 10)	4%	16%	S
Chloramphenicol (C 30)	16%	16%	I
Gentamicin (GEN 10)	26%	14%	S
Colistin/sulphamethane (CL 10)	18%	6%	S
Neomycin (N 10)	8%	34%	S
Flumequine (UB 30)	12%	12%	S
Enrofloxacin (EX 5)	0%	28%	S
Ciprofloxacin (CIP 5)	0%	18%	S
Imipenem (IPM 10)	0%	4%	S
Cefoperazone/sulbactam (CES 105)	0%	2%	S

R, resistant; I, Intermediate resistance, S, Sensitive.

**Table 4:** Antimicrobial resistance genes carried on plasmids recovered from human and poultry *Salmonella* serovars.

Human Serovars, Code	Antimicrobial Resistance Genes Detected	Poultry Serovars, Code	Antimicrobial Resistance Genes Detected
*S.* Typhimurium, H2	*bla*_CTX-m_, *bla*_SHV_	*S.* Typhimurium, A52	*bla*_CTX-m_, *bla*_SHV_
*S.* Typhimurium, H3	*bla*_CTX-m_, *bla*_TEM_	*S.* Agama, A63	*bla*_CTX-m_
*S.* Typhimurium, H4	*bla*_CTX-m_, *bla*_SHV_	*S.* Virchow, A75	*bla*_CTX-m_, *bla*_TEM_
*S.* Typhimurium, H5	*bla*_CTX-m_, *bla*_TEM_	*S.* Sanktjohan	*bla*_CTX-m_, *bla*_SHV_
*S.* Enteritidis, H11	*bla*_CTX-m_, *bla*_SHV_	*S.* Stratford, A78	*bla*_CTX-m_, *bla*_SHV_
*S.* Enteritidis, H12	*bla*_CTX-m_, *bla*_TEM_	*S.* Mississippi, A79	*bla*_CTX-m_
*S.* Enteritidis, H13	*bla*_CTX-m_, *bla*_TEM_	*S.* Papuana, A81	*bla*_CTX-m_, *bla*_SHV_
*S.* Lumberhurst, H22	*bla*_CTX-m_	*S.* Bonariensis, A83	*bla*_CTX-m_, *aac*(6′)-Ib
*S.* Tumodi, H28	*bla*_CTX-m_, *bla*_SHV_		
*S.* Tesive, H31	*bla*_CTX-m_, *aac*(6′)-Ib		
*S.* Butontan, H36	*bla*_CTX-m_, *bla*_SHV_, *bla*_TEM_		

*bla*_TEM_: gene coding for TEM extended spectrum β-lactamase; *bla*_SHV_: gene coding for SHV extended spectrum β-lactamase; *aac*(6’)-Ib: gene coding for aminoglycoside 6’-N-acetyltransferase type Ib ciprofloxacin resistant variant; *bla*_CTX-m_: gene coding for cefotaxime (CTX-M) extended spectrum β-lactamase.

**Table 5:** Virulence genotypes (*n* = 11) detected among human *Salmonella* serovars (*n* = 50).

Virulence Genotypes	Number of Serovars	Codes of Serovars
*inv*A	4	H8, H17, H21, H47
*inv*A, *pef*A, *spv*C	1	H12
*inv*A, s*pi*A	4	H1, H19, H26, H30
*inv*A, s*pi*A*, pef*A	1	H15
*inv*A, s*pi*A, *spv*C	3	H4, H7, H35
*inv*A, s*pi*A, *stn*	2	H3, H44
*inv*A, s*pi*A, *stn*, *pef*A	10	H9, H22, H23, H24
*inv*A, s*pi*A, *stn*, *pef*A, *spv*C	3	H2, H5, H20
*inv*A, *spv*C	14	H11, H13, H14, H25
*inv*A, *stn*	3	H10, H16, H38
*inv*A, *stn*, *pef*A	5	H31, H37, H6, H18

**Table 6:** Virulence genotypes (*n* = 12) detected among poultry *Salmonella* serovars (*n* = 50).

Virulence Genotypes	Number of Serovars	Codes of Serovars
*inv*A	4	A94, A95, A98, A99
*inv*A, s*pi*A	2	A52, A92
*inv*A, s*pi*A, *spv*C	6	A63, A66, A73, A76, A78, A83
*inv*A, s*pi*A, *stn*	11	A51, A53, A55, A57, A59, A61, A65, A68, A71, A80, A96
*inv*A, s*pi*A, *stn*, *pef*A	19	A54, A56, A58, A60, A62, A64, A67, A69, A70, A72, A74, A76, A81, A82, A84, A85, A86, A97, A100
*inv*A, s*pi*A, *stn*, *pef*A, *spv*C	2	A79, A93
*inv*A, s*pi*A, *stn*, *spv*C	1	A89
*inv*A, *spv*C	1	A75
*inv*A, *stn*	1	A87
*inv*A, *stn*, *pef*A	1	A91
*inv*A, *stn*, *pef*A, *spv*C	1	A88
*inv*A, *stn*, *spv*C	1	A90

**Table 7:** Prevalence of virulence genes of among human and poultry *Salmonella* serovars.

Virulence Genes	Human Serovars	Poultry Serovars	Standard Strain
*Inv*A	100%	100%	+
*Spi*A	46%	82%	+
*Stn*	46%	74%	+
*Pef*A	40%	48%	+
*Spv*C	42%	24%	+

+ detected via PCR.

**Table 8:** Similarity index (Jaccard/Tanimoto Coefficient and number of intersecting elements) between the 24 human and poultry serovars using the online tool (https://planetcalc.com/1664/) accessed on 5 April 2021. V1 (*S.* Typhimurium H4), V2 (*S.* Enteritidis H14), V3 (*S.* Lumberhurst H23), V4 (*S.* Tumodi H28), V5 (*S.* Tesive H32), V6 (*S.* Anatum H38), V7 (*S.* Taksony H42), V8 (*S.* Hull H43), V9 (*S.* Agama H45), V10 (*S.* Dublin H48), V11 (*S.* Blegdam H49), V12 (*S.* Butontan H35), V13 (*S.* Chester A71), V14 (*S.* Infantis A72), V15 (*S.* Kentucky A67), V16 (*S.* Virchow A75), V17 (*S.* Newport A85), V18 (*S.* Nitra (dog) A80), V19 (*S.* Montevideo A88), V20 (*S.* Gueuletapee A92), V21 (*S.* Kottbus A95), V22 (*S.* Sekondi A97), V23 (*S.* Newlands A100), V24 (*S.* Gallinarum A98).

		Jaccard/Tanimoto Coefficient																							
		1	2	3	4	5	6	7	8	9	10	11	12	13	14	15	16	17	18	19	20	21	22	23	24
**No of Intersecting Elements**	**1**		0.33	1	0.67	0.33	0.67	0.33	0.67	0.17	0.33	0.17	0.17	0.33	0.33	0.5	0.33	0.5	0.33	0.5	0.17	0.33	0.33	0.17	0.33
	**2**	2		0.33	0.5	1	0.5	1	0.5	0.5	1	0.5	0.5	1	1	0.67	1	0.67	1	0.67	0.5	1	1	0.5	1
	**3**	6	2		0.67	0.33	0.67	0.33	0.67	0.17	0.33	0.17	0.17	0.33	0.33	0.5	0.33	0.5	0.33	0.5	0.17	0.33	0.33	0.17	0.33
	**4**	4	2	4		0.5	1	0.5	1	0.25	0.5	0.25	0.25	0.5	0.5	0.75	0.5	0.75	0.5	0.75	0.25	0.5	0.5	0.25	0.5
	**5**	2	2	2	2		0.5	1	0.5	0.5	1	0.5	0.5	1	1	0.67	1	0.67	1	0.67	0.5	1	1	0.5	1
	**6**	4	2	4	4	2		0.5	1	0.25	0.5	0.25	0.25	0.5	0.5	0.75	0.5	0.75	0.5	0.75	0.25	0.5	0.5	0.25	0.5
	**7**	2	2	2	2	2	2		0.5	0.5	1	0.5	0.5	1	1	0.67	1	0.67	1	0.67	0.5	1	1	0.5	1
	**8**	4	2	4	4	2	4	2		0.25	0.5	0.25	0.25	0.5	0.5	0.75	0.5	0.75	0.5	0.75	0.25	0.5	0.5	0.25	0.5
	**9**	1	1	1	1	1	1	1	1		0.5	1	1	0.5	0.5	0.33	0.5	0.33	0.5	0.33	1	0.5	0.5	1	0.5
	**10**	2	2	2	2	2	2	2	2	1		0.5	0.5	1	1	0.67	1	0.67	1	0.67	0.5	1	1	0.5	1
	**11**	1	1	1	1	1	1	1	1	1	1		1	0.5	0.5	0.33	0.5	0.33	0.5	0.33	1	0.5	0.5	1	0.5
	**12**	1	1	1	1	1	1	1	1	1	1	1		0.5	0.5	0.33	0.5	0.33	0.5	0.33	1	0.5	0.5	1	0.5
	**13**	2	2	2	2	2	2	2	2	1	2	1	1		1	0.67	1	0.67	1	0.67	0.5	1	1	0.5	1
	**14**	2	2	2	2	2	2	2	2	1	2	1	1	2		0.67	1	0.67	1	0.67	0.5	1	1	0.5	1
	**15**	3	2	3	3	2	3	2	3	1	2	1	1	2	2		0.67	1	0.67	1	0.33	0.67	0.67	0.33	0.67
	**16**	2	2	2	2	2	2	2	2	1	2	1	1	2	2	2		0.67	1	0.67	0.5	1	1	0.5	1
	**17**	3	2	3	3	2	3	2	3	1	2	1	1	2	2	3	2		0.67	1	0.33	0.67	0.67	0.33	0.67
	**18**	2	2	2	2	2	2	2	2	1	2	1	1	2	2	2	2	2		0.67	0.5	1	1	0.5	1
	**19**	3	2	3	3	2	3	2	3	1	2	1	1	2	2	3	2	2	2		0.33	0.67	0.67	0.33	0.67
	**20**	1	1	1	1	1	1	1	1	1	1	1	1	1	1	1	1	1	1	1		0.5	0.5	1	0.5
	**21**	2	2	2	2	2	2	2	2	1	2	1	1	2	2	2	2	2	2	2	1		1	0.5	1
	**22**	2	2	2	2	2	2	2	2	1	2	1	1	2	2	2	2	2	2	2	1	2		0.5	1
	**23**	1	1	1	1	1	1	1	1	1	1	1	1	1	1	1	1	1	1	1	1	1	1		0.5
	**24**	2	2	2	2	2	2	2	2	1	2	1	1	2	2	2	2	2	2	2	1	2	2	1	

**Table 9:** Oligonucleotide primers sequences of tested virulence genes.

Target Gene	Primer	Primer Sequence (5′ → 3′)	Product Size (bp)	Ta * (°C)	Reference
*inv*A	*inv*A-F	GTGAAATTATCGCCACGTTCGGGCAA	284	55	[39]
	*inv*A-R	TCATCGCACCGTCAAAGGAACC			
*spi*A	*spi*A-F	GGTTCCAGGGGTCGTTAGTG	308	55	This study
	*spi*A-R	ACGCAATTTTCTTGCCACCC			
*spv*C	*spv*C-F	TGCACAACCAAATGCGGAAG	584	55	This study
	*spv*C-R	CGGTTCCTCACGTAAAGCCT			
*stn*	*stn*-F	CAGCCCTTTGATGGACGAGA	223	55	This study
	*stn*-R	CGACACAGGCAAGGGAGTAA			
*pef*A	*pef*A-F	TGAAAAAGAGCATTATTGCTTCCA	378	54	This study
	*pef*A-R	CACCAGGATTGGTTTTTGCGT			
*bla*_CTX-m_	*ctx-F*	CGCTTTGCGATGTGCAG	550	47	[40]
	*ctx-R*	ACCGCGATATCGTTGGT			
*bla*_TEM,_	*tem-F*	ATGAGTATTCAACATTTCCG	867	47	[41]
	*tem-R*	CTGACAGTTACCAATGCTTA			
*bla*_SHV_	*SHV-F*	GGTTATGCGTTATATTCGCC	867	47	[41]
	*SHV-R*	TTAGCGTTGCCAGTGCTC			
*aac*(6′)-Ib	*aac-F*	TTGCGATGCTCTATGAGTGG	358	46	[42]
	*aac-R*	CGTTTGGATCTTGGTGACCT			

* Ta: annealing temperature; *inv*A: encoding invasion protein; *spi*A: encoding type 3 secretion system secretin; *spv*C: encoding secreted effector protein (*Salmonella* plasmid virulence); *stn*: encoding heat-labile *Salmonella* enterotoxin; *pef*A: encoding plasmid-encoded fimbrial protein; *bla*_TEM_: gene coding for TEM extended spectrum β-lactamase; *bla*_SHV_: gene coding for SHV extended spectrum β-lactamase; *aac*(6′)-Ib: gene coding for aminoglycoside 6′-N-acetyltransferase type Ib ciprofloxacin resistant variant; *bla*_CTX-m_: gene coding for cefotaxime (CTX-M) extended spectrum β-lactamase.

**Table 10:** Human and poultry serovars selected for ERIC PCR.

Human Serovar	ERIC-PCR Code	Code	Poultry Serovar	ERIC-PCR Code	Code
*S.* Typhimurium	1	H4	*S.* Chester	13	A71
*S.* Enteritidis	2	H14	*S.* Infantis	14	A72
*S.* Lumberhurst	3	H23	*S.* Kentucky	15	A67
*S.* Tumodi	4	H28	*S.* Virchow	16	A75
*S.* Tesive	5	H32	*S.* Newport	17	A85
*S.* Anatum	6	H38	*S.* Nitra (dog)	18	A80
*S.* Taksony	7	H42	*S.* Montevideo	19	A88
*S.* Hull	8	H43	*S.* Gueuletapee	20	A92
*S.* Agama	9	H45	*S.* Kottbus	21	A95
*S.* Dublin	10	H48	*S.* Sekondi	22	A97
*S.* Blegdam	11	H49	*S.* Newlands	23	A100
*S.* Butontan	12	H35	*S.* Gallinarum	24	A98

**Table 11:** Oligonucleotide sequences of ERIC Primer.

Primer	Primer Sequence (5’ → 3’)	Product Size (bp)	Ta * (°C)	Reference
Forward	GTGAAATTATCGCCACGTTCGGGCAA	variable	52	[43]
Reverse	TCATCGCACCGTCAAAGGAACC			

* Ta: annealing temperature, ERIC: encoding enterobacterial repetitive intergenic consensus.

## Data Availability

Data are available within the article and Supplementary Materials.

## Supplementary Materials

The following are available online at https://www.mdpi.com/article/10.3390/antibiotics10050493/s1, Table S1: Antibiogram analysis of the 50 human *Salmonella* serovars against the 28 tested antimicrobial agents, Table S2: Antibiogram analysis of the 50 poultry *Salmonella* serovars against the 28 tested antimicrobial agents, Table S3: Prevalence of MDR isolates among human, poultry serovars and the standard strain, Table S4: Virulence genes and plasmid profile analysis (PPA) of the 50 human *Salmonella* serovars, Table S5: Virulence genes and plasmid profile analysis (PPA) of the 50 poultry *Salmonella* serovars, Figure S1A–C: Plasmid profile analysis of different ampicillin resistant Salmonella serovars isolates, Figure S2: Agarose gel electrophoresis of PCR products of *inv*A gene (284 bp) of some selected isolates, Figure S3: Agarose gel electrophoresis of PCR products of *spi*A gene (308 bp) of some selected isolates, Figure S4: Agarose gel electrophoresis of PCR products of *stn* gene (223 bp) and *pef*A gene (378 bp) of some selected isolates, Figure S5: Agarose gel electrophoresis of PCR products of *spv*C gene (584 bp) of some selected isolates, Figure S6: ERIC-PCR of the 24 (12 human and 12 poultry) serovars.
