# Supplementary material for: Serotyping and Antimicrobial Resistance Profile of Enteric Nontyphoidal Salmonella Recovered from Febrile Neutropenic Patients and Poultry in Egypt
Source: Antibiotics (Basel). 2021 Apr 26;10(5):493. doi: 10.3390/antibiotics10050493 (PMC8147078; doi:10.3390/antibiotics10050493)
Supplement: Supplementary file 1 [file antibiotics-10-00493-s001.zip › antibiotics-1196656-supplementary.pdf]

Table S1: Antibiogram analysis of the 50 human *Salmonella* serovars against the 28 tested antimicrobial agents

Antimicrobial agents	Antibiotic disc	Isolate codes of *Salmonella* Typhimurium Serovars									
		H1	H2	H3	H4	H5	H6	H7	H8	H9	H10
Cefradine	CH 30	R	R	R	R	R	R	R	R	R	R
Clindamycin	CD2	R	R	R	R	R	R	R	R	R	R
Nalidixic acid	NA 30	I	S	I	S	I	I	I	S	I	I
Erythromycin	E 15	R	R	R	R	R	R	R	R	R	R
Ciprofloxacin	CIP 5	S	S	S	S	S	S	S	S	S	S
Trimoxazole/sulphmethoprim	COT 25	S	R	S	S	S	S	S	S	S	S
Norfloxacin	NX 10	S	S	S	S	S	S	S	S	S	S
Rifampicin	RIF 5	R	R	R	R	R	R	R	R	R	R
Spiramycin	SR 100	I	R	R	R	R	R	R	R	I	I
Enrofloxacin	EX 5	I	S	S	S	S	S	S	S	I	S
Chloramphenicol	C 30	S	I	S	S	R	S	R	S	S	I
Gentamicin	GEN 10	S	S	S	S	R	S	R	S	S	S
Colistin/sulphamethane	CL 10	S	S	S	S	S	S	S	S	S	S
Neomycin	N 10	S	S	S	I	I	S	I	I	S	S
Streptomycin	S 10	R	R	I	I	I	I	I	I	R	S
Cefotaxime	CTX 30	R	R	R	R	R	R	R	R	R	R
Doxycycline	DO 30	R	I	I	I	I	I	I	I	R	I
Amoxicillin	AMX 10	R	R	R	R	R	R	R	R	R	R
Oxytetracycline	O 30	R	R	I	I	I	I	I	I	R	R
Flumequine	UB 30	S	S	R	S	R	R	R	S	S	S
Lincomycin	L 2	R	R	R	R	R	R	R	R	R	R
Nitrofurantoin	F 300	R	I	R	I	R	R	R	I	R	I
Tobramycin.	TOB 10	R	R	R	R	R	R	R	R	R	R
Cefoperazone/sulbactam	CES 105	S	S	S	S	S	S	S	S	S	I
Imipenem	IPM 10	S	S	S	S	S	S	S	S	S	S
Ampicillin/sulbactam	SAM 20	R	R	R	R	R	R	R	R	S	R
Amoxicillin/clavulanic acid	AMC30	R	R	R	R	R	R	R	R	R	R
Ampicillin	AMP 10	R	R	R	R	R	R	R	R	R	S

Continue (Table S1.)

Antimicrobial agents	Antibiotic disc	Isolate codes of *S.* Enteritidis Serovars								S. Lumberhurst	
		H11	H12	H13	H14	H15	H16	H17	H18	H19	H20
Cefradine	CH 30	R	R	I	R	R	R	R	R	I	R
Clindamycin	CD2	R	R	R	R	R	R	R	R	R	R
Nalidixic acid	NA 30	S	S	S	R	R	R	S	R	I	R
Erythromycin	E 15	R	R	R	R	R	R	R	R	R	R
Ciprofloxacin	CIP 5	S	S	S	S	S	S	S	S	S	S
Trimoxazole/sulphmethoprim	COT 25	S	S	R	S	R	S	S	S	S	S
Norfloxacin	NX 10	S	S	S	S	S	S	S	S	S	S
Rifampicin	RIF 5	R	R	R	R	R	R	R	R	R	R
Spiramycin	SR 100	R	R	S	R	R	R	R	R	I	I
Enrofloxacin	EX 5	S	S	S	S	I	S	S	S	I	S
Chloramphenicol	C 30	S	S	S	S	S	S	S	S	I	S
Gentamicin	GEN 10	S	S	S	R	R	R	S	R	S	R
Colistin/sulphamethane	CL 10	S	S	S	R	S	R	S	R	R	S
Neomycin	N 10	I	I	S	R	R	R	I	R	S	I
Streptomycin	S 10	S	S	S	R	I	R	S	R	I	I
Cefotaxime	CTX 30	R	R	R	R	R	R	R	R	R	R
Doxycycline	DO 30	R	R	R	R	R	R	R	R	R	R
Amoxicillin	AMX 10	R	R	R	R	R	R	R	R	R	R
Oxytetracycline	O 30	R	R	R	R	S	R	R	R	R	R
Flumequine	UB 30	S	S	S	I	S	I	S	I	S	S
Lincomycin	L 2	R	R	R	R	R	R	R	R	R	R
Nitrofurantoin	F 300	I	I	R	R	I	R	I	R	R	R
Tobramycin.	TOB 10	R	R	R	R	R	R	R	R	R	R
Cefoperazone/sulbactam	CES 105	S	S	S	I	S	I	S	I	S	S
Imipenem	IPM 10	S	S	S	S	S	S	S	S	S	S
Ampicillin/sulbactam	SAM 20	R	R	R	R	R	R	R	R	R	R
Amoxicillin/clavulanic acid	AMC30	R	R	R	R	R	R	R	R	R	R
Ampicillin	AMP 10	R	R	R	R	R	R	R	R	R	R

Continue (Table S1.)

Antimicrobial agents	Antibiotic disc	Isolate codes *S.* Lumberhurst serovars						Isolate codes *S.* Tumodi serovars			
		H21	H22	H23	H24	H25	H26	H27	H28	H29	H30
Cefradine	CH 30	I	R	I	R	I	R	R	R	R	R
Clindamycin	CD2	R	R	R	R	R	R	R	R	R	R
Nalidixic acid	NA 30	I	R	I	R	I	R	S	I	I	S
Erythromycin	E 15	R	R	R	R	R	R	R	R	R	R
Ciprofloxacin	CIP 5	S	S	S	S	S	S	S	S	S	S
Trimoxazole/sulphmethoprim	COT 25	S	S	S	S	S	S	S	I	S	S
Norfloxacin	NX 10	S	S	S	S	S	S	S	S	S	S
Rifampicin	RIF 5	R	R	R	R	R	R	R	R	R	R
Spiramycin	SR 100	I	I	I	I	I	I	R	R	R	R
Enrofloxacin	EX 5	I	S	I	S	I	S	S	S	S	I
Chloramphenicol	C 30	I	S	I	S	I	S	S	R	I	S
Gentamicin	GEN 10	S	R	S	R	S	R	S	S	S	S
Colistin/sulphamethane	CL 10	R	S	R	S	R	S	S	S	S	S
Neomycin	N 10	S	I	S	I	S	I	S	S	I	S
Streptomycin	S 10	I	I	I	I	I	I	I	I	R	I
Cefotaxime	CTX 30	R	R	R	R	R	R	R	R	R	R
Doxycycline	DO 30	R	R	R	R	R	R	I	I	I	I
Amoxicillin	AMX 10	R	R	R	R	R	R	R	R	R	R
Oxytetracycline	O 30	R	R	R	R	R	R	I	R	R	I
Flumequine	UB 30	S	S	S	S	S	S	S	R	R	S
Lincomycin	L 2	R	R	R	R	R	R	R	R	R	R
Nitrofurantoin	F 300	R	R	R	R	R	R	S	R	S	R
Tobramycin.	TOB 10	R	R	R	R	R	R	R	R	R	R
Cefoperazone/sulbactam	CES 105	S	S	S	S	S	S	S	S	S	S
Imipenem	IPM 10	S	S	S	S	S	S	S	S	S	S
Ampicillin/sulbactam	SAM 20	R	R	R	R	R	R	R	R	R	R
Amoxicillin/clavulanic acid	AMC30	R	R	R	R	R	R	R	R	R	R
Ampicillin	AMP 10	R	R	R	R	R	R	R	R	R	R

Continue (Table S1.)

Antimicrobial agents	Antibiotic disc	Isolate Codes *S.* Tesive serovars				Isolate Codes *S.* Butontan			Isolate Codes *S.* Antum		
		H31	H32	H33	H34	H35	H36	H37	H38	H39	H40
Cefradine	CH 30	R	I	R	I	R	R	R	R	R	R
Clindamycin	CD2	R	R	R	R	R	R	R	R	R	R
Nalidixic acid	NA 30	I	S	I	S	S	I	S	I	I	I
Erythromycin	E 15	R	R	R	R	R	R	R	R	R	R
Ciprofloxacin	CIP 5	S	S	S	S	S	S	S	S	S	S
Trimoxazole/sulphmethoprim	COT 25	S	S	S	S	S	S	S	S	S	S
Norfloxacin	NX 10	S	S	S	S	S	S	S	S	S	S
Rifampicin	RIF 5	R	R	R	R	R	R	R	R	R	R
Spiramycin	SR 100	I	I	I	I	R	R	R	I	I	I
Enrofloxacin	EX 5	S	S	S	S	S	S	S	S	S	S
Chloramphenicol	C 30	S	R	S	R	S	R	S	I	I	I
Gentamicin	GEN 10	I	S	I	S	S	R	I	S	S	S
Colistin/sulphamethane	CL 10	S	S	S	S	S	S	S	S	S	S
Neomycin	N 10	I	S	I	S	I	I	I	I	I	I
Streptomycin	S 10	R	R	R	R	S	R	I	R	R	R
Cefotaxime	CTX 30	R	R	R	R	R	R	R	R	R	R
Doxycycline	DO 30	I	R	I	R	S	R	R	R	R	R
Amoxicillin	AMX 10	R	R	R	R	R	R	R	R	R	R
Oxytetracycline	O 30	S	I	S	I	S	R	I	R	R	R
Flumequine	UB 30	S	S	S	S	S	S	S	S	S	S
Lincomycin	L 2	R	R	R	R	R	R	R	R	R	R
Nitrofurantoin	F 300	R	I	R	I	I	I	I	R	R	R
Tobramycin.	TOB 10	R	R	R	R	R	R	R	R	R	R
Cefoperazone/sulbactam	CES 105	S	S	S	S	S	S	S	S	S	S
Imipenem	IPM 10	S	S	S	S	S	S	S	S	S	S
Ampicillin/sulbactam	SAM 20	R	R	R	R	R	R	R	R	R	R
Amoxicillin/clavulanic acid	AMC30	R	R	R	R	R	R	R	R	R	R
Ampicillin	AMP 10	R	R	R	R	R	R	R	R	R	R

Continue (Table S1.)

Antimicrobial agents	Antibiotic disc	H41	H42	H43	H44	H45	H46	H47	H48	H49	H50
		*S.*Taksony	*S.*Taksony	*S.*Hull	*S.*Hull	*S.*Agama	*S.*Agama	*S.*Dublin	*S.*Dublin	*S.*Blegdam	*S.*Blegdam
Cefradine	CH 30	R	R	I	R	R	R	R	R	R	R
Clindamycin	CD2	R	R	R	R	R	R	R	R	R	R
Nalidixic acid	NA 30	I	I	S	S	I	I	I	I	S	S
Erythromycin	E 15	R	R	R	R	R	R	R	R	R	R
Ciprofloxacin	CIP 5	S	S	S	S	S	S	S	S	S	S
Trimoxazole/sulphmethoprim	COT 25	I	I	S	S	S	S	S	S	I	I
Norfloxacin	NX 10	R	R	S	S	S	S	S	S	S	S
Rifampicin	RIF 5	R	R	R	R	R	R	R	R	R	R
Spiramycin	SR 100	R	R	I	R	I	I	I	I	R	R
Enrofloxacin	EX 5	I	I	S	S	S	S	S	S	I	I
Chloramphenicol	C 30	R	R	S	S	I	I	I	I	S	S
Gentamicin	GEN 10	S	S	R	R	S	S	S	S	S	S
Colistin/sulphamethane	CL 10	R	R	S	S	S	S	S	S	S	S
Neomycin	N 10	I	I	S	I	S	S	I	I	I	I
Streptomycin	S 10	I	I	R	R	S	S	R	R	R	R
Cefotaxime	CTX 30	R	R	R	R	R	R	R	R	R	R
Doxycycline	DO 30	R	R	R	R	I	I	R	R	R	R
Amoxicillin	AMX 10	R	R	R	R	R	R	R	R	R	R
Oxytetracycline	O 30	R	R	I	S	R	R	R	R	R	R
Flumequine	UB 30	S	S	S	S	S	S	S	S	S	S
Lincomycin	L 2	R	R	R	R	I	I	R	R	I	I
Nitrofurantoin	F 300	R	R	I	I	I	I	R	R	I	I
Tobramycin.	TOB 10	R	R	R	R	R	R	R	R	R	R
Cefoperazone/sulbactam	CES 105	S	S	S	S	S	S	S	S	S	S
Imipenem	IPM 10	S	S	S	S	S	S	S	S	S	S
Ampicillin/sulbactam	SAM 20	R	R	R	R	R	R	R	R	R	R
Amoxicillin/clavulanic acid	AMC30	R	R	R	R	R	R	R	R	R	R
Ampicillin	AMP 10	R	R	R	R	R	R	R	R	R	R

Table S2: Antibiogram analysis of the 50 poultry *Salmonella* serovars against the 28 tested antimicrobial agents

Antimicrobial agents	Antibiotic disc	*S.* Typhimurium	*S.* Typhimurium	*S.* Enteritidis	*S.* Enteritidis	*S.* Enteritidis	*S.* Enteritidis	*S.* Enteritidis	*S.* Lumberhurst	*S.* Tsevie	*S.* Butontan
		A51	A52	A53	A54	A55	A56	A57	A58	A59	A60
Cefradine	CH 30	R	R	R	I	R	I	R	R	R	R
Clindamycin	CD2	R	R	R	R	R	R	R	R	R	R
Nalidixic acid	NA 30	S	S	S	S	S	S	S	S	S	R
Erythromycin	E 15	R	R	R	R	R	R	R	R	R	R
Ciprofloxacin	CIP 5	S	S	S	S	S	S	S	S	S	S
Trimoxazole/sulphmethoprim	COT 25	S	I	S	R	R	R	R	S	S	I
Norfloxacin	NX 10	S	S	S	S	S	S	S	R	S	S
Rifampicin	RIF 5	R	R	R	R	R	R	R	R	R	R
Spiramycin	SR 100	R	R	R	I	R	I	R	R	R	R
Enrofloxacin	EX 5	S	S	S	S	S	S	S	S	S	S
Chloramphenicol	C 30	S	S	S	S	S	S	S	S	I	S
Gentamicin	GEN 10	S	S	I	S	S	S	S	I	R	S
Colistin/sulphamethane	CL 10	S	S	S	S	S	S	S	S	S	S
Neomycin	N 10	S	I	R	S	S	S	S	R	R	I
Streptomycin	S 10	I	R	R	R	S	R	S	I	I	R
Cefotaxime	CTX 30	I	R	R	R	R	R	R	R	I	R
Doxycycline	DO 30	R	R	R	R	R	R	R	R	R	R
Amoxicillin	AMX 10	R	R	R	R	R	R	R	R	R	R
Oxytetracycline	O 30	R	R	R	R	R	R	R	R	R	R
Flumequine	UB 30	S	I	S	I	S	I	S	S	S	S
Lincomycin	L 2	R	R	R	R	R	R	R	R	R	R
Nitrofurantoin	F 300	R	R	R	R	R	R	R	R	R	R
Tobramycin.	TOB 10	R	R	R	R	R	R	R	R	R	R
Cefoperazone/sulbactam	CES 105	S	S	S	S	S	S	S	S	S	S
Imipenem	IPM 10	S	S	S	S	S	S	S	S	S	S
Ampicillin/sulbactam	SAM 20	R	R	R	R	R	R	R	R	R	R
Amoxicillin/clavulanic acid	AMC30	R	R	R	R	R	R	R	R	R	R
Ampicillin	AMP 10	R	R	R	R	R	R	R	R	R	R

Continue (Table S2)

Antimicrobial agents	Antibiotic disc	*S.* Anat	*S.* Anat	*S.* Agama	*S.* Taksony	*S.* Bleg	*S.* Bleg	*S.* Kent	*S.* Kent	*S.* Bou	*S.* Bou
		A61	A62	A63	A64	A65	A66	A67	A68	A69	A70
Cefradine	CH 30	R	R	R	R	R	I	R	R	R	R
Clindamycin	CD2	R	R	R	R	R	R	R	R	R	R
Nalidixic acid	NA 30	I	I	I	R	S	S	R	R	R	R
Erythromycin	E 15	R	R	R	R	R	R	R	R	R	R
Ciprofloxacin	CIP 5	S	S	I	R	S	S	R	R	R	R
Trimoxazole/sulphmethoprim	COT 25	S	S	R	R	S	R	R	R	R	R
Norfloxacin	NX 10	S	S	S	R	S	S	R	R	R	R
Rifampicin	RIF 5	R	R	R	R	R	R	R	R	R	R
Spiramycin	SR 100	R	R	R	I	R	I	R	R	R	R
Enrofloxacin	EX 5	I	I	S	R	S	S	S	S	R	R
Chloramphenicol	C 30	I	I	S	R	S	S	R	R	R	R
Gentamicin	GEN 10	S	S	S	S	I	S	R	R	I	I
Colistin-sulphamethane	CL 10	R	R	I	R	S	S	S	S	S	S
Neomycin	N 10	I	I	I	R	R	S	R	R	R	I
Streptomycin	S 10	I	I	I	R	R	R	R	R	I	I
Cefotaxime	CTX 30	R	R	R	R	R	R	R	R	R	R
Doxycycline	DO 30	R	R	R	R	R	R	R	R	R	R
Amoxicillin	AMX 10	R	R	R	R	R	R	R	R	R	R
Oxytetracycline	O 30	R	R	R	R	R	R	R	R	R	R
Flumequine	UB 30	S	S	R	R	S	I	S	S	I	I
Lincomycin	L 2	R	R	R	R	R	R	R	R	R	R
Nitrofurantoin	F 300	R	R	R	R	R	R	R	R	R	R
Tobramycin.	TOB 10	R	R	R	R	R	R	R	R	R	R
Cefoperazone/sulbactam	CES 105	S	S	S	S	S	S	S	S	S	S
Imipenem	IPM 10	R	R	S	S	S	S	S	S	S	S
Ampicillin/sulbactam	SAM 20	R	R	R	R	R	R	I	I	R	R
Amoxicillin/clavulanic acid	AMC30	I	I	R	R	R	R	S	S	R	R
Ampicillin	AMP 10	R	R	R	R	R	R	R	R	R	R

Continue (Table S2)

Antimicrobial agents	Antibiotic disc	*S.* Chest	*S.* Infant	*S.* Fur	*S.* Crem	*S.* Virch	*S.* Kedo	*S.* Sank	*S.* Stratford	*S.* Missi	*S.* Nitra
		A71	A72	A73	A74	A75	A76	A77	A78	A79	80
Cefradine	CH 30	R	R	R	I	R	I	R	R	R	R
Clindamycin	CD2	R	R	R	R	R	R	R	R	R	R
Nalidixic acid	NA 30	R	R	S	R	S	S	I	S	S	R
Erythromycin	E 15	R	R	R	R	R	R	R	R	R	R
Ciprofloxacin	CIP 5	R	S	S	S	S	S	S	S	S	S
Trimoxazole/sulphmethoprim	COT 25	I	R	S	R	S	S	I	S	S	I
Norfloxacin	NX 10	R	S	S	S	S	S	S	S	S	S
Rifampicin	RIF 5	R	R	R	R	R	R	R	R	R	R
Spiramycin	SR 100	I	R	R	I	R	I	R	R	R	R
Enrofloxacin	EX 5	R	S	S	R	S	S	S	S	I	S
Chloramphenicol	C 30	R	S	I	R	S	S	S	S	S	S
Gentamicin	GEN 10	S	S	S	S	S	I	R	R	S	S
Colistin-sulphamethane	CL 10	S	S	S	S	S	S	S	S	S	S
Neomycin	N 10	S	I	R	S	S	S	S	R	R	I
Streptomycin	S 10	I	R	S	R	R	I	R	R	I	R
Cefotaxime	CTX 30	R	R	R	R	R	R	R	R	I	R
Doxycycline	DO 30	R	R	R	R	R	I	R	R	I	R
Amoxicillin	AMX 10	R	R	R	R	R	R	R	R	R	R
Oxytetracycline	O 30	R	R	R	R	R	R	R	R	R	R
Flumequine	UB 30	R	R	S	R	S	I	S	S	S	S
Lincomycin	L 2	R	R	R	R	R	R	R	R	R	R
Nitrofurantoin	F 300	R	R	R	R	R	R	R	R	R	R
Tobramycin.	TOB 10	R	R	R	R	R	R	R	R	R	R
Cefoperazone/sulbactam	CES 105	I	S	S	R	S	I	S	S	S	S
Imipenem	IPM 10	S	S	S	S	S	S	S	S	S	S
Ampicillin/sulbactam	SAM 20	R	R	R	R	R	R	R	R	R	R
Amoxicillin/clavulanic acid	AMC30	R	R	R	R	R	R	R	R	R	R
Ampicillin	AMP 10	R	R	R	R	R	R	R	R	R	R

Continue (Table S2)

Antimicrobial agents	Antibiotic disc	*S.* Papu	*S.* Bardo	*S.* Bona	*S.* Boeck	*S.* Newport	*S.* Colin	*S.* Hato	*S.* Mont	*S.* Farsta	*S.* Krali
		A81	A82	A83	A84	A85	A86	A87	A88	A89	A90
Cefradine	CH 30	R	R	R	I	R	R	R	R	R	R
Clindamycin	CD2	R	R	R	R	R	R	R	R	R	R
Nalidixic acid	NA 30	R	S	R	R	S	S	R	S	S	R
Erythromycin	E 15	R	R	R	R	R	R	R	R	R	R
Ciprofloxacin	CIP 5	S	S	R	R	S	S	S	S	S	S
Trimoxazole/sulphmethoprim	COT 25	S	I	S	S	R	S	R	S	S	I
Norfloxacin	NX 10	S	S	S	S	S	S	S	S	S	S
Rifampicin	RIF 5	R	R	R	R	R	R	R	R	R	R
Spiramycin	SR 100	R	R	R	I	R	I	R	R	R	R
Enrofloxacin	EX 5	I	S	R	S	S	S	I	S	S	S
Chloramphenicol	C 30	S	S	I	I	S	I	S	I	I	S
Gentamicin	GEN 10	R	S	S	S	S	S	I	I	R	S
Colistin-sulphamethane	CL 10	S	S	S	S	S	S	S	S	S	S
Neomycin	N 10	S	I	R	S	S	S	R	S	R	I
Streptomycin	S 10	I	R	R	R	S	I	S	I	I	R
Cefotaxime	CTX 30	R	R	R	R	R	R	R	R	I	R
Doxycycline	DO 30	R	R	R	R	R	R	R	R	R	R
Amoxicillin	AMX 10	R	R	R	R	R	R	R	R	R	R
Oxytetracycline	O 30	R	R	R	R	R	R	R	R	R	R
Flumequine	UB 30	I	I	S	R	S	S	S	I	S	S
Lincomycin	L 2	R	R	R	R	R	R	R	R	R	R
Nitrofurantoin	F 300	R	R	R	R	R	I	R	R	R	R
Tobramycin.	TOB 10	R	R	R	R	R	R	R	R	R	R
Cefoperazone/sulbactam	CES 105	S	S	S	S	S	S	S	S	S	S
Imipenem	IPM 10	S	S	S	S	S	S	S	S	S	S
Ampicillin/sulbactam	SAM 20	R	R	R	R	R	R	R	R	R	R
Amoxicillin/clavulanic acid	AMC30	R	R	R	R	R	R	R	R	R	R
Ampicillin	AMP 10	R	R	R	R	R	R	R	S	R	R

Continue (Table S2)

Antimicrobial agents	Antibiotic disc	*S.* Fill	*S.* Gueul	*S.* Atak	*S.* Stanly	*S.* Kottbus	*S.* Magher	*S.* Sekon	*S.* Gall	*S.* Volta	*S.* Newlands
		A91	A92	A93	A94	A95	A96	A97	A98	A99	A100
Cefradine	CH 30	R	R	R	I	R	I	I	R	R	R
Clindamycin	CD2	R	R	R	R	R	R	R	R	R	R
Nalidixic acid	NA 30	S	S	S	S	S	S	R	S	S	R
Erythromycin	E 15	R	R	R	R	R	R	R	R	R	R
Ciprofloxacin	CIP 5	S	S	S	S	S	S	R	S	S	S
Trimoxazole/sulphmethoprim	COT 25	S	I	S	R	R	R	R	S	S	I
Norfloxacin	NX 10	S	S	S	S	S	S	I	R	S	S
Rifampicin	RIF 5	R	R	R	R	R	R	R	R	R	R
Spiramycin	SR 100	R	R	R	I	R	I	I	R	R	R
Enrofloxacin	EX 5	S	S	S	S	S	S	R	S	S	S
Chloramphenicol	C 30	S	S	S	S	S	S	R	S	S	S
Gentamicin	GEN 10	S	S	I	S	S	S	S	I	S	S
Colistin/sulphamethane	CL 10	S	S	S	S	S	S	S	S	S	S
Neomycin	N 10	S	I	R	S	S	S	R	R	I	I
Streptomycin	S 10	I	R	R	R	S	R	I	I	I	R
Cefotaxime	CTX 30	I	R	R	R	R	R	R	R	R	R
Doxycycline	DO 30	R	R	R	R	R	R	R	R	I	R
Amoxicillin	AMX 10	R	R	R	R	R	R	R	R	R	R
Oxytetracycline	O 30	R	R	R	R	R	R	R	R	S	R
Flumequine	UB 30	S	I	S	I	S	I	S	S	S	S
Lincomycin	L 2	R	R	R	R	R	R	R	R	R	R
Nitrofurantoin	F 300	R	R	R	R	R	R	I	R	R	R
Tobramycin.	TOB 10	R	R	R	R	R	R	R	R	R	R
Cefoperazone/sulbactam	CES 105	S	S	S	S	S	S	S	S	S	S
Imipenem	IPM 10	S	S	S	S	S	S	S	S	S	S
Ampicillin/sulbactam	SAM 20	R	R	R	R	R	R	R	R	R	R
Amoxicillin/clavulanic acid	AMC30	R	R	R	R	R	R	R	R	R	R
Ampicillin	AMP 10	R	R	R	R	R	R	R	R	R	R

Table S3: Prevalence of MDR isolates among human, poultry serovars and the standard strain.

Code	Serovar	Ampicillin	Oxytetracycline	Erythromycin	Trimoxazole/sulphmethoprim	Chloramphenicol	Cefotaxime	Ciprofloxacin	No. of R
		AMP 10	O 30	E 15	COT 25	C 30	CTX 30	CIP 5	
H1	*S.* Typhimurium	R	R	R	S	S	R	S	4
H2		R	R	R	R	I	R	S	5
H3		R	I	R	S	S	R	S	3
H4		R	I	R	S	S	R	S	3
H5		R	I	R	S	R	R	S	4
H6		R	I	R	S	S	R	S	3
H7		R	I	R	S	R	R	S	4
H8		R	I	R	S	S	R	S	3
H9		R	R	R	S	S	R	S	4
H10		S	R	R	S	I	R	S	3
H11	*S.* Enteritidis	R	R	R	S	S	R	S	4
H12		R	R	R	S	S	R	S	4
H13		R	R	R	R	S	R	S	5
H14		R	R	R	S	S	R	S	4
H15		R	S	R	R	S	R	S	4
H16		R	R	R	S	S	R	S	4
H17		R	R	R	S	S	R	S	4
H18		R	R	R	S	S	R	S	4
H19	*S.* Lumberhurst	R	R	R	S	I	R	S	4
H20		R	R	R	S	S	R	S	4
H21		R	R	R	S	I	R	S	4
H22		R	R	R	S	S	R	S	4
H23		R	R	R	S	I	R	S	4
H24		R	R	R	S	S	R	S	4
H25		R	R	R	S	I	R	S	4
H26		R	R	R	S	S	R	S	4
H27	*S.* Tumodi	R	I	R	S	S	R	S	3
H28		R	R	R	I	R	R	S	5
H29		R	R	R	S	I	R	S	4
H30		R	I	R	S	S	R	S	3
H31	*S.* Tesive	R	S	R	S	S	R	S	3
H32		R	I	R	S	R	R	S	4
H33		R	S	R	S	S	R	S	3
H34		R	I	R	S	R	R	S	4
H35	*S.* Butontan	R	S	R	S	S	R	S	3
H36		R	R	R	S	R	R	S	5
H37		R	I	R	S	S	R	S	3
H38	*S.* Antum	R	R	R	S	I	R	S	4

H39		R	R	R	S	I	R	S	4
H40		R	R	R	S	I	R	S	4
H41	*S.* Taksony	R	R	R	I	R	R	S	5
H42		R	R	R	I	R	R	S	5
H43	*S.* Hull	R	I	R	S	S	R	S	3
H44		R	S	R	S	S	R	S	3
H45	*S.* Agama	R	R	R	S	I	R	S	4
H46		R	R	R	S	I	R	S	4
H47	*S.* Dublin	R	R	R	S	I	R	S	4
H48		R	R	R	S	I	R	S	4
H49	*S.* Blegdam	R	R	R	I	S	R	S	4
H50		R	R	R	I	S	R	S	4
STD	*S.* Poona standard	R	R	R	S	I	R	S	4
A51	*S.* Typhimurium	R	R	R	S	S	I	S	3
A52		R	R	R	I	S	R	S	4
A53	*S.* Enteritidis	R	R	R	S	S	R	S	4
A54		R	R	R	R	S	R	S	4
A55		R	R	R	R	S	R	S	5
A56		R	R	R	R	S	R	S	5
A57		R	R	R	R	S	R	S	5
A58	*S.* Lumberhurst	R	R	R	S	S	R	S	4
A59	*S.* Tsevie	R	R	R	S	I	I	S	3
A60	*S.* Butontan	R	R	R	I	S	R	S	4
A61	*S.* Anatum	R	R	R	S	I	R	S	4
A62		R	R	R	S	I	R	S	4
A63	*S.* Agama	R	R	R	R	S	R	I	5
A64	*S.* Taksony	R	R	R	R	R	R	R	7
A65	*S.* Blegdam	R	R	R	S	S	R	S	4
A66		R	R	R	R	S	R	S	5
A67	*S.* Kentucky	R	R	R	R	R	R	R	7
A68		R	R	R	R	R	R	R	7
A69	*S.* Bouake	R	R	R	R	R	R	R	7
A70		R	R	R	R	R	R	R	7
A71	*S.* Chester	R	R	R	I	R	R	R	6
A72	*S.* Infantis	R	R	R	R	S	R	S	5
A73	*S.* Furuch	R	R	R	S	I	R	S	4
A74	*S.* Cremieu	R	R	R	R	R	R	S	6
A75	*S.* Virchow	R	R	R	S	S	R	S	4
A76	*S.* Kedougou	R	R	R	S	S	R	S	4
A77	*S.* Sanktjohan	R	R	R	I	S	R	S	4
A78	*S.* Stratford	R	R	R	S	S	R	S	4
A79	*S.* Mississippi	R	R	R	S	S	I	S	3

A80	*S.* Nitra (dog)	R	R	R	I	S	R	S	4
A81	*S.* Papuana	R	R	R	S	S	R	S	4
A82	*S.* Bardo	R	R	R	I	S	R	S	4
A83	*S.* Bonariensis	R	R	R	S	I	R	R	5
A84	*S.* Boecker	R	R	R	S	I	R	R	5
A85	*S.* Newport	R	R	R	R	S	R	S	5
A86	*S.* Colindal	R	R	R	S	I	R	S	4
A87	*S.* Hato	R	R	R	R	S	R	S	5
A88	*S.* Montevideo	S	R	R	S	I	R	S	3
A89	*S.* Farsta	R	R	R	S	I	I	S	3
A90	*S.* Kralingen	R	R	R	I	S	R	S	4
A91	*S.* Fillmore	R	R	R	S	S	I	S	3
A92	*S.* Gueuletapee	R	R	R	I	S	R	S	4
A93	*S.* Atakpame	R	R	R	S	S	R	S	4
A94	*S.* Stanly	R	R	R	R	S	R	S	5
A95	*S.* Kottbus	R	R	R	R	S	R	S	5
A96	*S.* Magherafelt	R	R	R	R	S	R	S	5
A97	*S.* Sekondi	R	R	R	R	R	R	R	7
A98	*S.* Gallinarum	R	R	R	S	S	R	S	4
A99	*S.* Volta	R	S	R	S	S	R	S	3
A100	*S.* Newlands	R	R	R	I	S	R	S	4

Table S4: Virulence genes and Plasmid Profile Analysis (PPA) of the 50 human *Salmonella* serovars.

Serovars	Code	*inv*A	*Spi*A	*stn*	*pef*A	*spv*C	PPA
S. Typhimurium	H1	+	+	-	-	-	-
	H2	+	+	+	+	+	+
	H3	+	+	+	-	-	+
	H4	+	+	-	-	+	+
	H5	+	+	+	+	+	+
	H6	+	-	+	+	-	NR
	H7	+	+	-	-	+	NR
	H8	+	-	-	-	-	NR
	H9	+	+	+	+	-	NR
	H10	+	-	+	-	-	NR
S. Enteritidis	H11	+	-	-	-	+	+
	H12	+	-	-	+	+	+
	H13	+	-	-	-	+	+
	H14	+	-	-	-	+	NR
	H15	+	+	-	+	-	NR
	H16	+	-	+	-	-	NR
	H17	+	-	-	-	-	NR
	H18	+	-	+	+	-	NR
S. Lumberhurst	H19	+	+	-	-	-	NR
	H20	+	+	+	+	+	NR
	H21	+	-	-	-	-	NR
	H22	+	+	+	+	-	+
	H23	+	+	+	+	-	NR
	H24	+	+	+	+	-	NR
	H25	+	-	-	-	+	NR
	H26	+	+	-	-	-	NR
S. Tumodi	H27	+	-	-	-	+	NR
	H28	+	+	+	+	-	+
	H29	+	-	-	-	+	NR
	H30	+	+	-	-	-	NR

S. Tesive	H31	+	-	+	+	-	+
	H32	+	-	-	-	+	NR
	H33	+	-	-	-	+	NR
	H34	+	-	+	+	-	NR
S. Butontan	H35	+	+	-	-	+	NR
	H36	+	+	+	+	-	+
	H37	+	-	+	+	-	NR
S. Antum	H38	+	-	+	-	-	NR
	H39	+	+	+	+	-	NR
	H40	+	+	+	+	-	NR
S. Taksony	H41	+	-	-	-	+	NR
	H42	+	-	-	-	+	NR
S. Hull	H43	+	+	+	+	-	NR
	H44	+	+	+	-	-	NR
S. Agama	H45	+	+	+	+	-	NR
	H46	+	-	-	-	+	NR
S. Dublin	H47	+	-	-	-	-	NR
	H48	+	-	-	-	+	NR
S. Blegdam	H49	+	-	-	-	+	NR
	H50	+	-	-	-	+	NR

***+,detected, -, not detected, and NR, no plasmid DNA was detected of the ampicillin sensitive serovars. *invA*: encoding invasion protein, *spiA*: encoding type 3 secretion system secretin, *spvC*: encoding secreted effector protein (*Salmonella* plasmid virulence), *stn*: encoding heat-labile *Salmonella* enterotoxin, *pefA*: encoding plasmid-encoded fimbrial protein.**

Table S5: Virulence genes and Plasmid Profile Analysis (PPA) of the 50 poultry *Salmonella* serovars and *S.* Poona standard strain

Serovars	Code	*inv*A	*Spi*A	*Stn*	*pef*A	*spv*C	PPA
S. Typhimurium	A51	+	+	+	-	-	-
	A52	+	+	-	-	-	+
S. Enteritidis	A53	+	+	+	-	-	-
	A54	+	+	+	+	-	**NR**
	A55	+	+	+	-	-	**NR**
	A56	+	+	+	+	-	**NR**
	A57	+	+	+	-	-	**NR**
S. Lumberhurst	A58	+	+	+	+	-	**NR**
S. Tsevie	A59	+	+	+	-	-	**NR**
S. Butontan	A60	+	+	+	+	-	**NR**
S. Anatum	A61	+	+	+	-	-	**NR**
	A62	+	+	+	+	-	**NR**
S. Agama	A63	+	+	-	-	+	+
S. Taksony	A64	+	+	+	+	-	**NR**
S. Blegdam	A65	+	+	+	-	-	-
	A66	+	+	-	-	+	**NR**
S. Kentucky	A67	+	+	+	+	-	-
	A68	+	+	+	-	-	**NR**
S. Bouake	A69	+	+	+	+	-	**NR**
	A70	+	+	+	+	-	**NR**
S. Chester	A71	+	+	+	-	-	**NR**
S. Infantis	A72	+	+	+	+	-	-
S. Furuch	A73	+	+	-	-	+	-
S. Cremieu	A74	+	+	+	+	-	-
S. Virchow	A75	+	-	-	-	+	+
S. Kedougou	A76	+	+	+	+	-	**NR**
S. Sanktjohan	A77	+	+	-	-	+	+
S. Stratford	A78	+	+	-	-	+	+
S. Mississippi	A79	+	+	+	+	+	+
S. Nitra (dog)	A80	+	+	+	-	-	**NR**

S. Papuana	A81	+	+	+	+	-	+
S. Bardo	A82	+	+	+	+	-	-
S. Bonariensis	A83	+	+	-	-	+	+
S. Boecker	A84	+	+	+	+	-	**NR**
S. Newport	A85	+	+	+	+	-	**NR**
S. Colindal	A86	+	+	+	+	-	**NR**
S. Hato	A87	+	-	+	-	-	**NR**
S. Montevideo	A88	+	-	+	+	+	**NR**
S. Farsta	A89	+	+	+	-	+	**NR**
S. Kralingen	A90	+	-	+	-	+	**NR**
S. Fillmore	A91	+	-	+	+	-	**NR**
S. Gueuletapee	A92	+	+	-	-	-	-
S. Atakpame	A93	+	+	+	+	+	**NR**
S. Stanly	A94	+	-	-	-	-	**NR**
S. Kottbus	A95	+	-	-	-	-	**NR**
S. Magherafelt	A96	+	+	+	-	-	-
S. Sekondi	A97	+	+	+	+	-	**NR**
S. Gallinarum	A98	+	-	-	-	-	**NR**
S. Volta	A99	+	-	-	-	-	**NR**
S. Newlands	A100	+	+	+	+	-	**NR**
***S.* Poona standard**	**STD**	+	+	+	+	+	+

***+,detected, -, not detected, and NR, no plasmid DNA was detected of the ampicillin sensitive serovars. *invA*: encoding invasion protein, *spiA*: encoding type 3 secretion system secretin, *spvC*: encoding secreted effector protein (*Salmonella* plasmid virulence), *stn*: encoding heat-labile *Salmonella* enterotoxin, *pefA*: encoding plasmid-encoded fimbrial protein.**

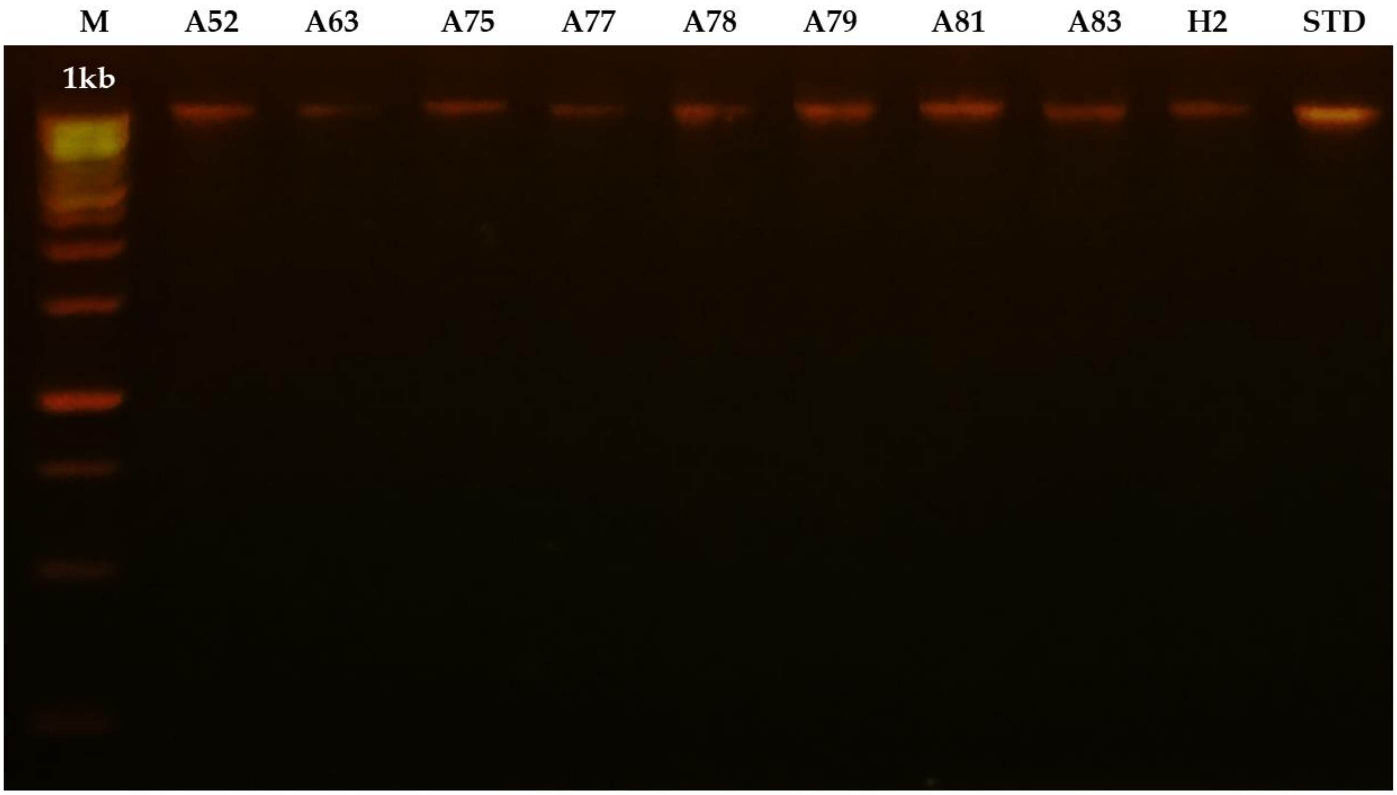

Figure S1A. Plasmid profile analysis of different ampicillin resistant *Salmonella* serovars isolates, and M, 1kb size marker. STD plasmid extracted from the *S.* Poona Standard serovar.

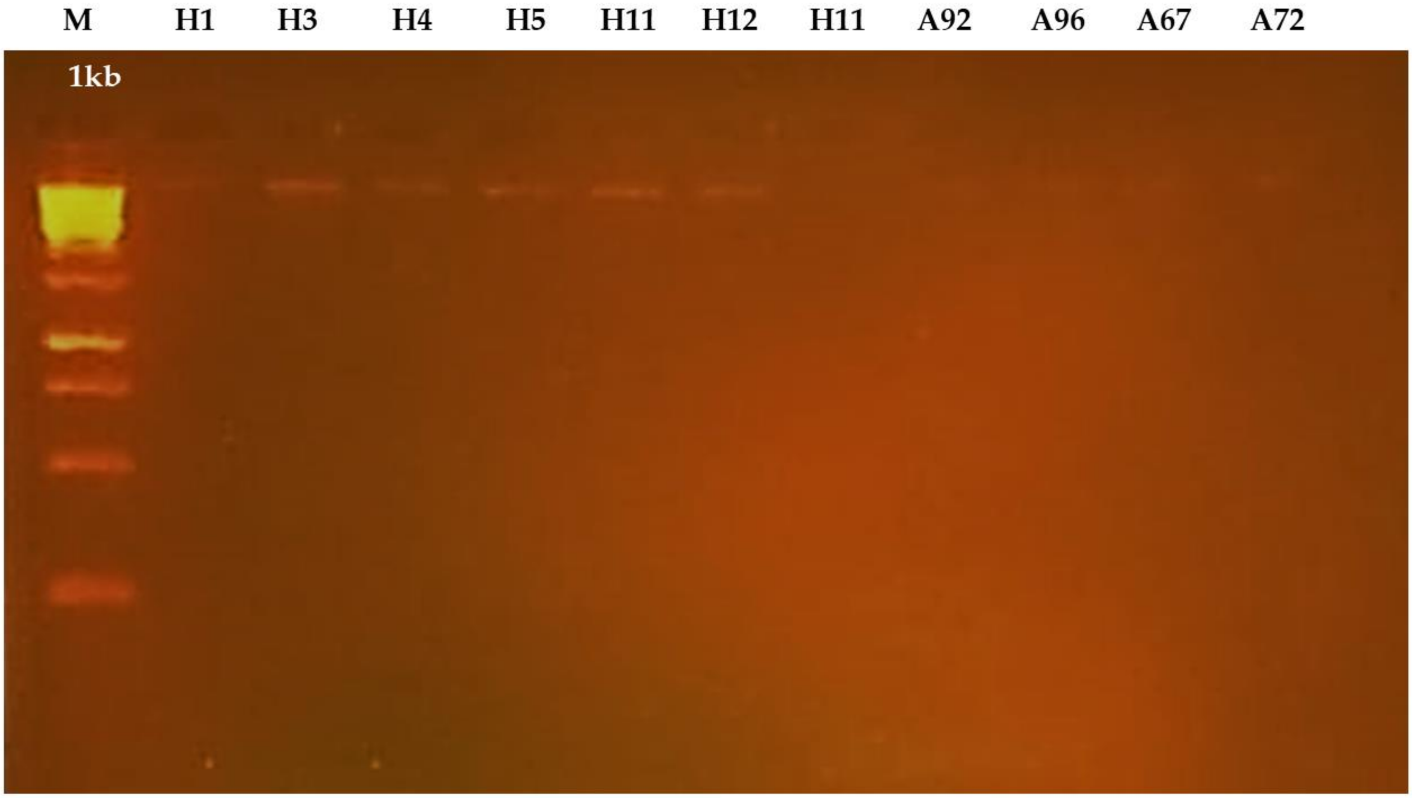

Figure S1B. Plasmid profile analysis of different ampicillin resistant isolates, and M, 1kb size marker.

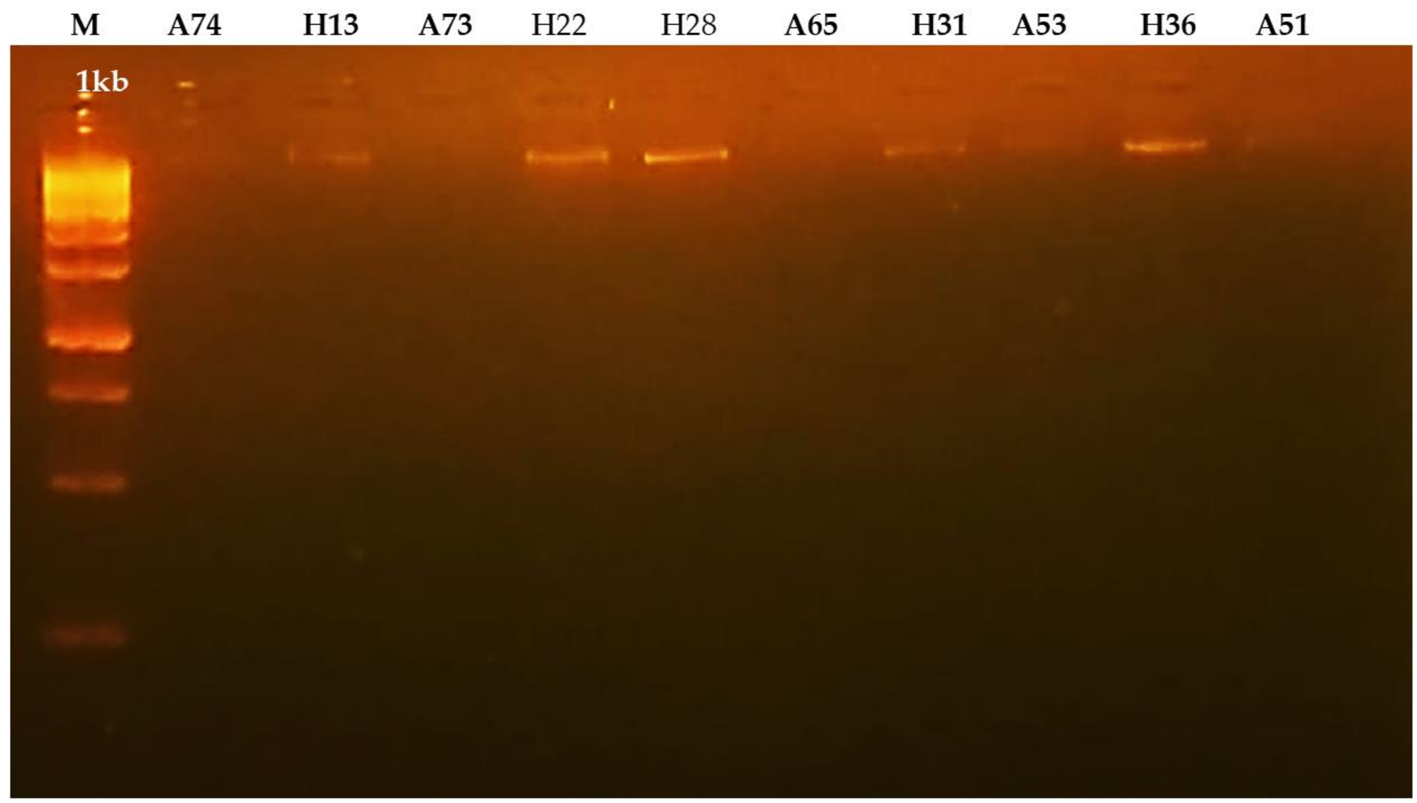

Figure S1C. Plasmid profile analysis of different ampicillin resistant isolates, and M, 1kb size marker.

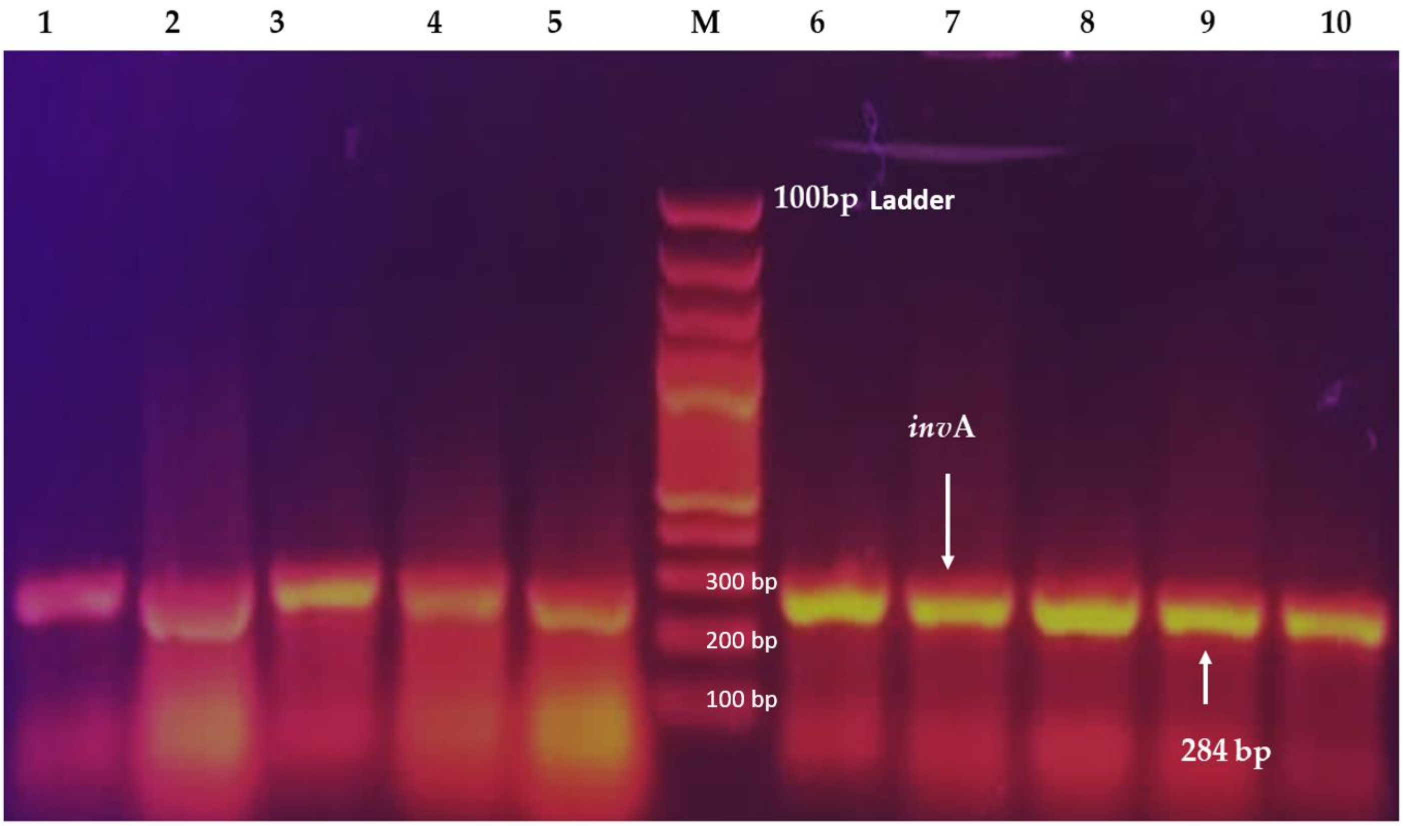

Figure S2. Agarose gel electrophoresis of PCR products of *inv*A gene (284 bp) of some selected isolates. Lanes: 1, *S.* Typhimurium; 2, *S.* Typhimurium; 3, *S.* Typhimurium; 4, *S.* Typhimurium; 5, *S.* Dublin; 6, *S.* Enteritidis; 7, *S.* Enteritidis; 8, *S.* Enteritidis; 9, *S.* Tesive; 10, *S.* Lumberhurst; and M, 100 bp size marker.

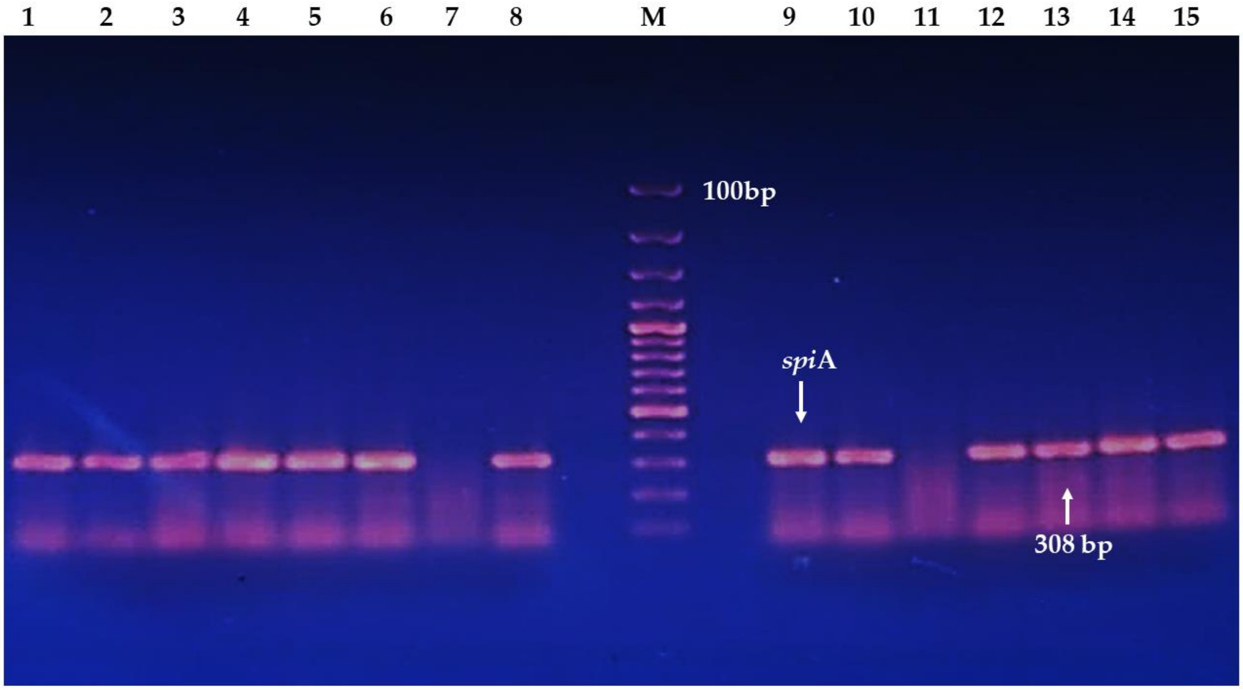

Figure S3. Agarose gel electrophoresis of PCR products of *spi*A gene (308 bp) of some selected isolates. Lanes: 1, *S.* Bonaresis; 2, *S.* Bardo; 3, *S.* Papuana; 4, *S.* Lumberhurst; 5, *S.* Typhimurium 15; 6, *S.* Lumberhurst; 7, *S.* Tumodi; 8, *S.* Enteritidis; 9, *S.* chester; 10, *S.* Typhimurium; 11, *S.* Tesive; 12, *S.* Typhimurium; 13, *S.* Typhimurium; 14, *S.* Lumberhurst; 15, *S.* Lumberhurst; and M, 100 bp size

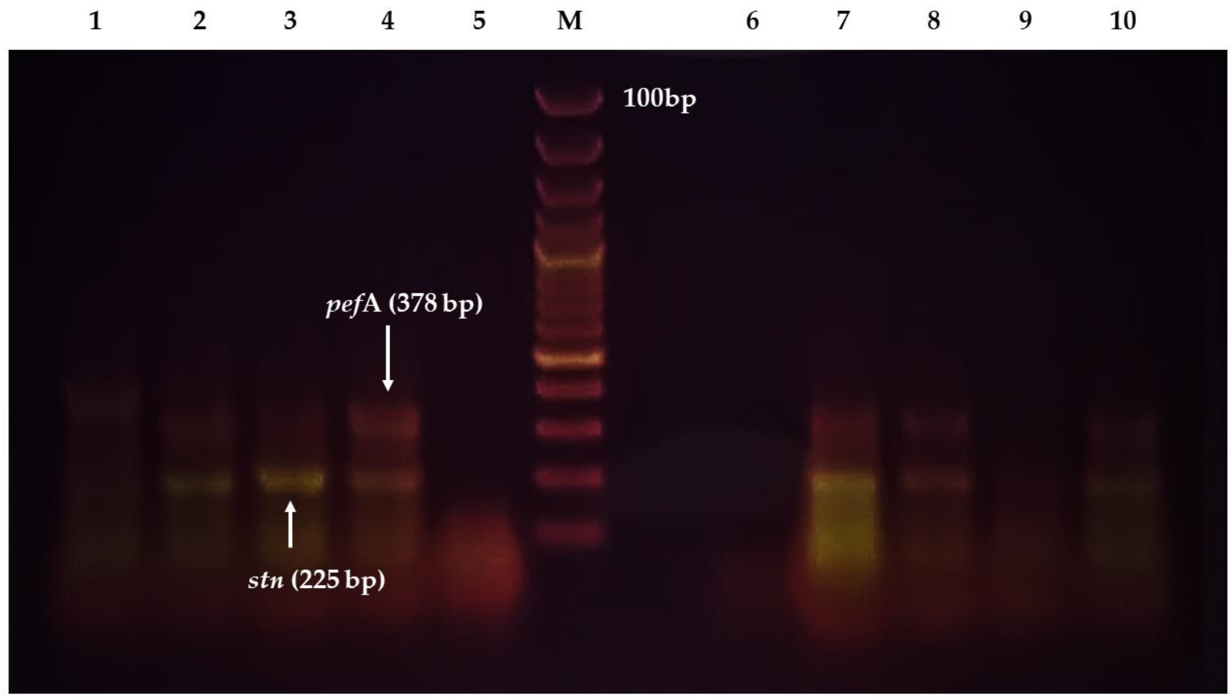

Figure S4. Agarose gel electrophoresis of PCR products of *stn* gene (223 bp) and *pef*A gene (378 bp) of some selected isolates. Lanes: 1, *S.* Bonaresis; 2, *S.* Bardo; 3, *S.* Papuana; 4, *S.* Lumberhurst; 5, *S.* Typhimurium; 6, *S.* Lumberhurst; 7, *S.* Tumodi; 8, *S.* Enteritidis; 9, *S.* chester; 10, *S.* Typhimurium; and M, 100 bp size

1 2 3 4 5 6 M 7 8 9 10 11

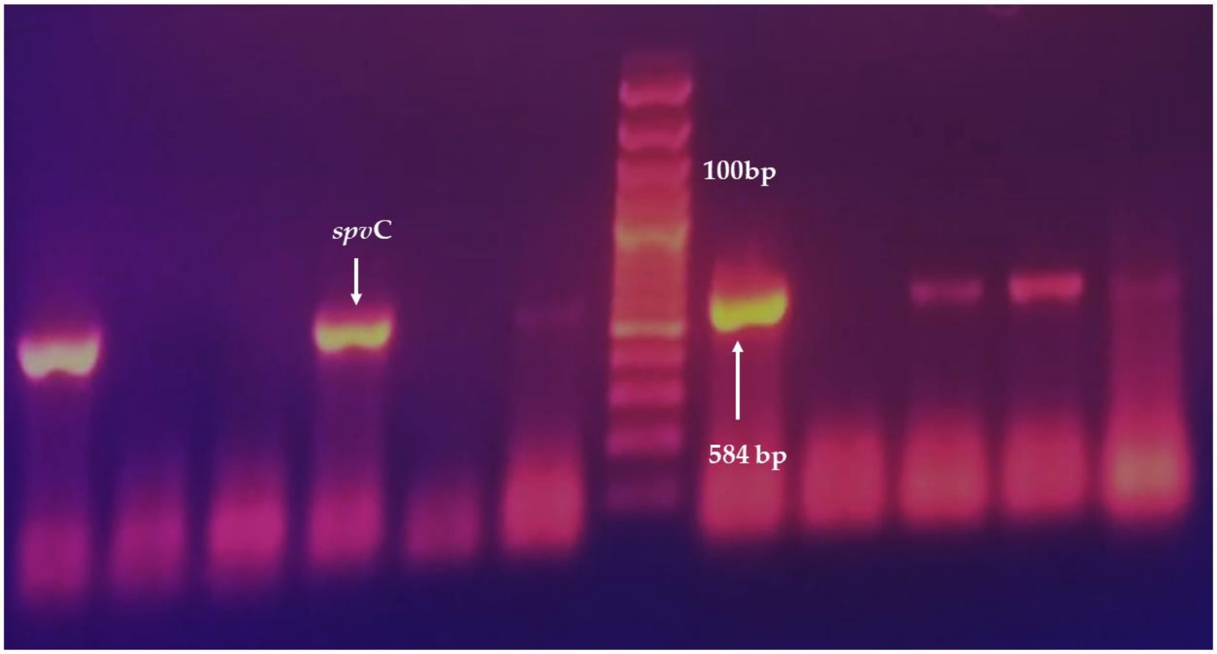

Figure S5. Agarose gel electrophoresis of PCR products of *spv*C gene (584 bp) of some selected isolates. Lanes: 1, *S.* Typhimurium; 2, *S.* Typhimurium; 3, *S.* Typhimurium; 4, *S.* Typhimurium; 5, *S.* Dublin; 6, *S.* Enteritidis; 7, *S.* Enteritidis; 8, *S.* Enteritidis; 9, *S.* Tesive; 10, *S.* Lumberhurst; 11, *S.* Tumodi; and M, 100 bp size marker

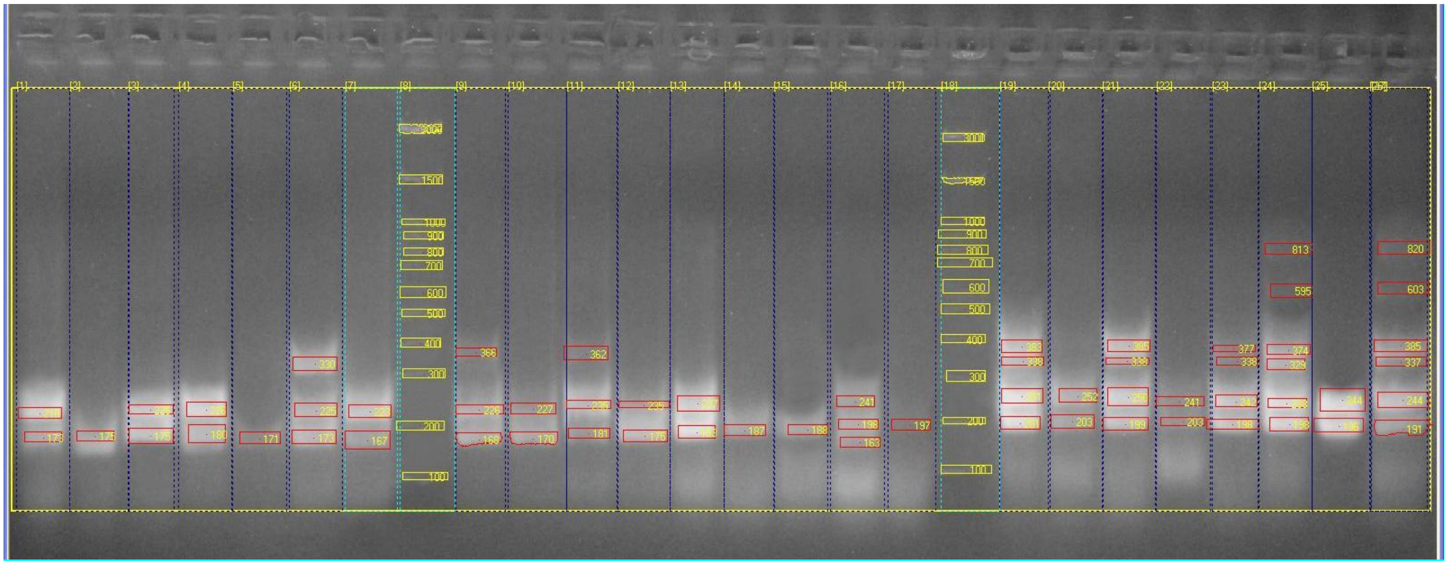

Figure S6. ERIC-PCR of the 24 (12 human and 12 poultry) serovars and L, 100 bp size ladder.
